# Comparative real-world progression free survival of CDK4/6 inhibitors in HR+/HER2− breast cancer patients with bone metastases

**DOI:** 10.1093/oncolo/oyag146

**Published:** 2026-04-16

**Authors:** Roberta Scafetta, Marco Donato, Carla Gullotta, Alessandra Guarino, Cristina Fiore, Luisana Sisca, Elena Speziale, Raffaella Troiano, Simone Foderaro, Fabio Venuti, Francesco Antonio Vilardi, Valentina Ricozzi, Michele Iuliani, Sonia Simonetti, Silvia Cavaliere, Alessio Cortellini, Annalisa La Cesa, Andrea Botticelli, Simone Scagnoli, Simona Pisegna, Carmen Criscitiello, Alessandra Chirco, Sara D’Alessandro, Rebecca Pedersini, Caterina Sposetti, Elisa Tiberi, Giuliana D’Auria, Matteo Vergati, Marco Mazzotta, Roberta Caputo, Annarita Verrazzo, Maria Grazia Rossino, Federica Domati, Claudia Piombino, Francesca Sofia Di Lisa, Lorena Filomeno, Teresa Arcuri, Federica Puce, Federica Riva, Michela Palleschi, Marianna Sirico, Marta Piras, Luigia Stefania Stucci, Delia De Lisi, Paolo Orsaria, Antonella Grasso, Edy Ippolito, Sara Ramella, Luca Visani, Niccolò Bertini, Ilaria Bonaparte, Stefania Gori, Luigi Rossi, Icro Meattini, Barbara Tagliaferri, Orazio Caffo, Maria Vittoria Bonomo, Ilaria Portarena, Azzurra Irelli, Elisabetta Cretella, Camillo Porta, Giampaolo Bianchini, Maria Agnese Fabbri, Ugo De Giorgi, Patrizia Vici, Angela Toss, Ornella Garrone, Michelino De Laurentiis, Federica Villa, Rossana Berardi, Mauro Minelli, Vittorio Altomare, Claudio Vernieri, Giuseppe Curigliano, Bruno Vincenzi, Giuseppe Tonini, Daniele Santini, Francesco Pantano

**Affiliations:** Medical Oncology, Fondazione Policlinico Universitario Campus Bio-Medico Via Alvaro del Portillo 200, Italy; Department of Medicine and Surgery, Università Campus Bio-Medico di Roma, Via Alvaro del Portillo 21, 00128, Roma, Italy; Division of Medical Oncology, “G.Martino” University Hospital, Via Consolare Valeria 1, 98124, Messina, Italy; Medical Oncology, Fondazione Policlinico Universitario Campus Bio-Medico Via Alvaro del Portillo 200, Italy; Medical Oncology, Fondazione Policlinico Universitario Campus Bio-Medico Via Alvaro del Portillo 200, Italy; Medical Oncology Unit, Santa Rosa Hospital, Department of Oncology and Hematology, Strada Sammartinese snc, 01100 Viterbo, Italy; Medical Oncology, Fondazione Policlinico Universitario Campus Bio-Medico Via Alvaro del Portillo 200, Italy; Medical Oncology, Fondazione Policlinico Universitario Campus Bio-Medico Via Alvaro del Portillo 200, Italy; Medical Oncology, Fondazione Policlinico Universitario Campus Bio-Medico Via Alvaro del Portillo 200, Italy; Department of Medicine and Surgery, Università Campus Bio-Medico di Roma, Via Alvaro del Portillo 21, 00128, Roma, Italy; Medical Oncology, Fondazione Policlinico Universitario Campus Bio-Medico Via Alvaro del Portillo 200, Italy; Medical Oncology, Fondazione Policlinico Universitario Campus Bio-Medico Via Alvaro del Portillo 200, Italy; Department of Medicine and Surgery, Università Campus Bio-Medico di Roma, Via Alvaro del Portillo 21, 00128, Roma, Italy; Medical Oncology, Fondazione Policlinico Universitario Campus Bio-Medico Via Alvaro del Portillo 200, Italy; Department of Medicine and Surgery, Università Campus Bio-Medico di Roma, Via Alvaro del Portillo 21, 00128, Roma, Italy; Medical Oncology, Fondazione Policlinico Universitario Campus Bio-Medico Via Alvaro del Portillo 200, Italy; Department of Medicine and Surgery, Università Campus Bio-Medico di Roma, Via Alvaro del Portillo 21, 00128, Roma, Italy; Medical Oncology, Fondazione Policlinico Universitario Campus Bio-Medico Via Alvaro del Portillo 200, Italy; Department of Medicine and Surgery, Università Campus Bio-Medico di Roma, Via Alvaro del Portillo 21, 00128, Roma, Italy; Medical Oncology, Fondazione Policlinico Universitario Campus Bio-Medico Via Alvaro del Portillo 200, Italy; Department of Medicine and Surgery, Università Campus Bio-Medico di Roma, Via Alvaro del Portillo 21, 00128, Roma, Italy; Medical Oncology, Fondazione Policlinico Universitario Campus Bio-Medico Via Alvaro del Portillo 200, Italy; Department of Radiological, Oncological and Pathological Science, "Sapienza" University of Rome, Rome, Italy; Department of Radiological, Oncological and Pathological Science, "Sapienza" University of Rome, Rome, Italy; Department of Experimental Medicine, Sapienza University, Rome, Italy; Medical Oncology Unit, Azienda Ospedaliero Universitaria Sant'Andrea, Via Di Grottarossa 1035-1039, Rome, Italy; Department of Oncology and Hemato-Oncology, University of Milan, Milano, Italy; ASST Papa Giovanni XXIII-SC Oncologia, 24127 Bergamo, Italy; ASST Papa Giovanni XXIII-SC Oncologia, 24127 Bergamo, Italy; Medical Oncology Department, ASST Spedali Civili of Brescia, Brescia, Italy; Department of Oncology and Hemato-Oncology, University of Milan, Milano, Italy; Dana-Farber/Partners CancerCare, Boston, MA, USA; Department of Medical Oncology, Fondazione IRCCS Istituto Nazionale dei Tumori, Milan, Italy; Department of Medical Oncology, Università Politecnica Delle Marche, AOU delle Marche, Ancona, Italy; Department of Medical Oncology, Medical Oncology Unit, Sandro Pertini Hospital, Rome, Italy; Department of Medical Oncology, Medical Oncology Unit, Sandro Pertini Hospital, Rome, Italy; Department of Medical Oncology, Medical Oncology Unit, Sandro Pertini Hospital, Rome, Italy; Department of Breast and Thoracic Oncology, Division of Breast Medical Oncology, Istituto di Ricovero e Cura a Carattere Scientifico (IRCCS) Pascale, Naples, Italy; Department of Breast and Thoracic Oncology, Division of Breast Medical Oncology, Istituto di Ricovero e Cura a Carattere Scientifico (IRCCS) Pascale, Naples, Italy; Medical Oncology Fondazione IRCCS Ca’ Granda Ospedale Maggiore Policlinico, 20122, Milan, Italy; Department of Oncology and Hematology, Azienda Ospedaliero-Universitaria di Modena, Modena, Italy; Department of Oncology and Hematology, Azienda Ospedaliero-Universitaria di Modena, Modena, Italy; UOSD Sperimentazioni di fase IV—IRCCS Istituto Nazionale Tumori Regina Elena, Via Elio Chianesi, Italia; UOSD Sperimentazioni di fase IV—IRCCS Istituto Nazionale Tumori Regina Elena, Via Elio Chianesi, Italia; UOSD Sperimentazioni di fase IV—IRCCS Istituto Nazionale Tumori Regina Elena, Via Elio Chianesi, Italia; Medical Oncology Unit, ICS Maugeri IRCCS, Pavia, Italy; Department of Clinical and Molecular Medicine, “La Sapienza” University of Rome Policlinico Umberto I, Viale Regina Elena 324, Italy; Medical Oncology, Breast & GYN Unit, IRCCS Istituto Romagnolo per lo Studio dei Tumori (IRST) “Dino Amadori”, Meldola, Italy; Medical Oncology, Breast & GYN Unit, IRCCS Istituto Romagnolo per lo Studio dei Tumori (IRST) “Dino Amadori”, Meldola, Italy; Department of Medical Oncology, IRCCS San Raffaele Scientific Institute, Milan, Italy; Interdisciplinary Department of Medicine, University of Bari “A. Moro” and Division of Medical Oncology, A.O.U. Consorziale Policlinico di Bari, Bari, Italy; Medical Oncology Unit, Santa Chiara Hospital, APSS Trento, Italy; Department of Breast Surgery, Fondazione Policlinico Universitario Campus Bio-Medico, Rome, Italy; Department of Breast Surgery, Fondazione Policlinico Universitario Campus Bio-Medico, Rome, Italy; Department of Radiation Oncology (Medicine and Surgery), Università Campus Bio-Medico di Roma, Rome, Italy; Radiation Oncology, Fondazione Policlinico Universitario Campus Bio-Medico, Rome, Italy; Department of Radiation Oncology (Medicine and Surgery), Università Campus Bio-Medico di Roma, Rome, Italy; Radiation Oncology, Fondazione Policlinico Universitario Campus Bio-Medico, Rome, Italy; Radiation Oncology Unit & Breast Unit, Azienda Ospedaliero-Universitaria Careggi, University of Florence, Florence, Italy; Medical Oncology, IRCCS Ospedale Sacro Cuore Don Calabria, Negrar di Valpolicella (VR), Italy; Radiation Oncology Unit & Breast Unit, Azienda Ospedaliero-Universitaria Careggi, University of Florence, Florence, Italy; Medical Oncology, IRCCS Ospedale Sacro Cuore Don Calabria, Negrar di Valpolicella (VR), Italy; UOC of Oncology - ASL Latina - Distretto 1, University of Rome "Sapienza", Formia, Italy; Radiation Oncology Unit & Breast Unit, Azienda Ospedaliero-Universitaria Careggi, University of Florence, Florence, Italy; Department of Experimental and Clinical Biomedical Sciences “M. Serio”, University of Florence, Florence, Italy; Department of Radiation Oncology (MAASTRO), GROW Research Institute for Oncology and Developmental Biology, Maastricht University Medical Centre, Maastricht, The Netherlands; Medical Oncology Unit, ICS Maugeri IRCCS, Pavia, Italy; Medical Oncology Unit, Santa Chiara Hospital, APSS Trento, Italy; Center of Medical Science – University of Trento, Italy; Medical Oncology Unit, Policlinico Universitario Tor Vergata, Italy; Medical Oncology Unit, Policlinico Universitario Tor Vergata, Italy; Medical Oncology Unit, Department of Oncology, "Giuseppe Mazzini" Hospital, AUSL 04 Teramo, 64100 Teramo, Italy; Medical Oncology Unit, Ospedale di Bolzano, Bolzano, Italia; Interdisciplinary Department of Medicine, University of Bari “A. Moro” and Division of Medical Oncology, A.O.U. Consorziale Policlinico di Bari, Bari, Italy; Department of Medical Oncology, IRCCS San Raffaele Scientific Institute, Milan, Italy; Università Vita-Salute San Raffaele, Milano, Italia; Medical Oncology Unit, Santa Rosa Hospital, Department of Oncology and Hematology, Strada Sammartinese snc, 01100 Viterbo, Italy; Department of Experimental Medicine, University of Salento, Lecce, Italy; UOSD Sperimentazioni di fase IV—IRCCS Istituto Nazionale Tumori Regina Elena, Via Elio Chianesi, Italia; Department of Oncology and Hematology, Azienda Ospedaliero-Universitaria di Modena, Modena, Italy; Department of Medical and Surgical Sciences, University of Modena and Reggio Emilia, Italy; Medical Oncology Fondazione IRCCS Ca’ Granda Ospedale Maggiore Policlinico, 20122, Milan, Italy; Department of Breast and Thoracic Oncology, Division of Breast Medical Oncology, Istituto di Ricovero e Cura a Carattere Scientifico (IRCCS) Pascale, Naples, Italy; Medical Oncology, Oncology Department ASST Lecco, 23900 Lecco, Italy; Department of Medical Oncology, Università Politecnica Delle Marche, AOU delle Marche, Ancona, Italy; UOC Oncologia Azienda Ospedaliera San Giovanni Addolorata, Rome, Italy; Department of Breast Surgery, Fondazione Policlinico Universitario Campus Bio-Medico, Rome, Italy; Department of Oncology and Hemato-Oncology, University of Milan, Milano, Italy; Department of Medical Oncology, Fondazione IRCCS Istituto Nazionale dei Tumori, Milan, Italy; Department of Oncology and Hemato-Oncology, University of Milan, Milano, Italy; European Institute of Oncology, IRCCS, Milano, Italy; Medical Oncology, Fondazione Policlinico Universitario Campus Bio-Medico Via Alvaro del Portillo 200, Italy; Department of Medicine and Surgery, Università Campus Bio-Medico di Roma, Via Alvaro del Portillo 21, 00128, Roma, Italy; Medical Oncology, Fondazione Policlinico Universitario Campus Bio-Medico Via Alvaro del Portillo 200, Italy; Department of Medicine and Surgery, Università Campus Bio-Medico di Roma, Via Alvaro del Portillo 21, 00128, Roma, Italy; Dip. Scienze e Biotecnologie Medico-chirurgiche, Policlinico Umberto I, "Sapienza" University of Rome, Rome, Italy; Medical Oncology, Fondazione Policlinico Universitario Campus Bio-Medico Via Alvaro del Portillo 200, Italy; Department of Medicine and Surgery, Università Campus Bio-Medico di Roma, Via Alvaro del Portillo 21, 00128, Roma, Italy

**Keywords:** breast cancer, bone metastases, CDK4/6 inhibitors

## Abstract

**Background:**

The introduction of CDK4/6 inhibitors (CDK4/6i) has improved outcomes in hormone receptor-positive (HR+)/HER2-negative metastatic breast cancer (mBC), including in patients with bone metastases. Assessing their comparative effectiveness in real-world settings is crucial.

**Methods:**

This retrospective, multicenter cohort study (January 2019–December 2023; median follow-up 39 months) evaluated the real-world progression-free survival (rwPFS) of abemaciclib, ribociclib, and palbociclib combined with endocrine therapy (ET) in HR+/HER2- mBC patients with bone metastases. Overall survival (OS) was a secondary exploratory endpoint. A total of 1399 patients with ECOG PS 0–1 and at least 12 months of follow-up were included. Analyses used propensity score matching (PSM) and inverse probability of treatment weighting (IPTW) to adjust for confounding.

**Results:**

Palbociclib showed shorter rwPFS (22 months) compared to abemaciclib (32 months; HR = 1.47, *p = *0.001) and ribociclib (35 months; HR = 1.49, *p < *0.001). No significant difference was observed between abemaciclib and ribociclib. OS was also lower with palbociclib (47 months) versus abemaciclib (60 months; HR = 1.77, *p < *0.001) and ribociclib (64 months; HR = 1.69, *p = *0.001). Results were consistent after PSM and IPTW adjustment.

**CONCLUSION:**

Ribociclib and abemaciclib may provide superior rwPFS and OS compared to palbociclib in HR+/HER2- mBC patients with bone metastases.

Implications for Practice The findings of this retrospective real-world study suggest that ribociclib and abemaciclib may be associated with longer progression-free survival and overall survival compared to palbociclib in patients with hormone receptor-positive, HER2-negative metastatic breast cancer with bone metastases. However, given the retrospective nature of the analysis and the potential for unmeasured confounding factors, these results should be interpreted with caution. These data can contribute to informed discussions between clinicians and patients when considering treatment options in everyday clinical practice

## Introduction

Breast cancer (BC) is the most prevalent malignancy worldwide and remains the leading cause of cancer-related deaths among women.^1^ Hormone receptor positive 2(HR+)/human epidermal growth factor receptor 2-negative (HER2-) BC, identified by positive immunohistochemical staining for the estrogen receptor (ER) and/or the progesterone receptor (PR), is the most diagnosed subtype.^2^ Significant advancements have been achieved in the treatment of HR+/Her2- metastatic BC (mBC) with the introduction of cyclin-dependent kinases4/6 inhibitors (CDK4/6i).^3^ These inhibitors work by targeting the cyclin D1-CDK4/6 holoenzyme, preventing the phosphorylation of the retinoblastoma protein. This effectively halts cell cycle progression at the G1 to S phase transition, impeding cancer cell proliferation.^4–7^ Additionally, blocking estrogen signaling pathways has been shown to reduce the activation of CDK4 and CDK6, reinforcing the effects of these inhibitors in controlling tumor growth.^8^

Currently, three CDK4/6i—abemaciclib, ribociclib, and palbociclib—have been approved by both the European Medicines Agency and the U.S. Food and Drug Administration for the treatment of mBC in combination with aromatase inhibitors (Ais) and fulvestrant. While all three drugs show similar benefits in terms of progression-free survival (PFS), ribociclib has the strongest evidence of overall survival (OS) benefit.^9–16^ The choice between these drugs is often guided by patient characteristics, side effect profiles, potential drug interactions, and the physician’s clinical experience.

Bone is the most frequent site for distant recurrence, occurring in around 40% of cases, and up to 80% of patients with mBC will develop bone metastases throughout their disease.^17–18^ Bone metastases can lead to skeletal-related events (SREs), which further impact quality of life and worsen the overall outcome.^19^ Subgroup analyses from pivotal trials, as well as meta-analyses, have shown the efficacy and safety of CDK4/6i even in patients with bone metastases.^9–16^

However, there is a lack of real-world evidence comparing the efficacy of these inhibitors specifically in patients with bone metastases. Our previous pre-clinical study revealed different effectiveness of palbociclib, abemaciclib, and ribociclib on the tumor bone microenvironment.^20^ This study aims to compare the real-world outcomes of abemaciclib, ribociclib, and palbociclib in HR+/HER2- mBC patients with bone metastases. By addressing the gaps in evidence from clinical trials and providing new insights from real-world data, this research seeks to optimize treatment strategies and improve patient outcomes in this specific population.

## Methods

### Study design

We retrospectively collected data from patients with HR+/HER2- mBC treated with CDK4/6i plus ET, as first or second line of treatment. Patients enrolled in the study had bone metastases at the time of metastatic disease diagnosis, an Eastern Cooperative Oncology Group performance status (ECOG PS) of 0 or 1, and a minimum follow-up period of 12 months. HER2-low patients were defined as those with an immunohistochemistry (IHC) score of 1+ or an IHC score of 2+ with a negative fluorescence in situ hybridization (FISH) result. Both endocrine-sensitive and endocrine-resistant patients were included. Definitions of endocrine- sensitivity and endocrine- resistance were as per the 4th ESO-ESMO International Consensus Guidelines for Advanced BC.^21^

Data collection spanned from January 2019 to December 2023. Ethical approval was granted by the ethics committee of the coordination center, Fondazione Policlinico Universitario Campus Bio-Medico (PAR 30.22 OSS). The study was conducted in accordance with the Declaration of Helsinki. Informed consent was obtained only from patients who were alive at the time of the analysis. The collection of data from deceased patients was authorized by the Data Protection Officer of the respective centers. The study population was identified through a review of medical records from the participating institutions.

### Statistical analysis

Given the retrospective observational design, sample size was determined by data availability and all eligible patients were included. In time-to-event analyses, statistical precision is primarily driven by the number of observed events; therefore, we evaluated the expected precision using an event-driven, post hoc assessment based on Schoenfeld’s approximation. Assuming a clinically meaningful effect size (hazard ratio = 1.4) and accounting for the three primary pairwise comparisons (Bonferroni-adjusted two-sided *α* = 0.0167), the observed rwPFS event counts were consistent with approximately >80% power for comparisons involving palbociclib, whereas power for the ribociclib vs abemaciclib comparison was lower due to the smaller abemaciclib group. For OS, event counts were lower and statistical power was correspondingly reduced; OS and subgroup analyses were therefore interpreted cautiously, reported with 95% confidence intervals, and considered exploratory.

To mitigate confounding effects and imbalanced clinicopathological characteristics among patients, we used propensity score matching (PSM) and the inverse probability of treatment weighting (IPTW) method. The PSM score was derived from logistic regression, with the CDK4/6i as the dependent variable and baseline variables as covariates. A 1:1 nearest neighbor matching with a caliper of 0.1 was implemented to establish matched cohorts. This caliper width is based on standard practices in statistical analysis, ensuring a balance between achieving a good match and retaining enough matched pairs for analysis.

IPTW methods assigned weights to patients, forming pseudo-populations where treatment allocation was independent of covariates. The probabilities of receiving a specific CDK4/6i were calculated using a logistic regression model, with CDK4/6i treatment as the outcome and baseline clinical characteristics as covariates. Individual weights were computed based on the inverse probabilities of receiving the assigned treatment.

Baseline patient characteristics were compared using the chi-square (*χ*^2^) and Fisher’s exact tests for categorical variables, and the t-test for continuous variables. The nonparametric Mann–Whitney U test was employed when the normality assumption was violated. The median follow-up time was estimated using the reverse Kaplan–Meier method.

Time-to-event outcomes (rwPFS and OS) were assessed using Kaplan–Meier estimates with log-rank tests and Cox proportional hazards models. Survival curves were generated using the Kaplan-Meier method and compared via the log-rank test for univariate analysis. Univariate Hazard Ratios (HRs) were computed using the log-rank method. A multivariable Cox regression model was employed to calculate HRs and 95% confidence intervals (CIs) for rwPFS and OS.

For the three pre-specified primary pairwise comparisons, we controlled multiplicity using Bonferroni correction (two-sided *α* = 0.0167). Subgroup and sensitivity analyses were exploratory and intended to assess consistency; thus, nominal *p*-values and 95% CIs are reported without formal multiplicity adjustment.

PSM, IPTW and Cox regression analysis were performed considering the following clinical variables: age, performance status, histology, Ki67 proliferative index, grading, ER expression, PR expression, HER2 status, neoadjuvant or adjuvant chemotherapy, adjuvant endocrine therapy, bone-only disease, number of bone metastases, visceral metastasis, endocrine sensitivity and endocrine therapy.

Sensitivity analysis was performed to ensure the robustness of our findings. We conducted Cox regression analyses for rwPFS and OS, both univariate and multivariate, by iteratively excluding patients from each enrolling institution. This approach allowed us to evaluate the impact of each center on the overall results.

To further assess the robustness of our findings against unmeasured confounding, we calculated the E-value for the observed HRs and their confidence intervals. The E-value quantifies the minimum strength of association that an unmeasured confounder would need to have with both the treatment and the outcome to explain away the observed association.^22^

Additional sensitivity analyses were conducted to address potential confounding by treatment line and calendar time. Specifically, rwPFS and OS analyses were repeated restricting the cohort to first-line CDK4/6i initiation only and, separately, to patients who initiated CDK4/6i therapy from 2020 onward. Cox models were also refit with adjustment for year of treatment initiation and treatment line where applicable.

Variables demonstrating statistical significance at a p value of less than 0.05 were included. PSM and IPTW analyses were conducted using the RStudio Addins and Shiny Modules for Medical Research package, as well as the Jamovi software suite, version 1.6.^23–24^

## Results

### Patient characteristics

We enrolled 1399 HR+/HER2- patients with mBC and bone metastases treated with ET plus CDK4/6i as first- or second-line of therapy. All patients had bone metastases at diagnosis of metastatic disease. Of these, 786 patients (56%) received palbociclib, 394 (28%) received ribociclib, and 219 (16%) were treated with abemaciclib. All patients’ clinical features are shown in Table 1. The variables Ki-67, ER, PR, and the number of bone metastases were dichotomized into high and low categories based on their median values: Ki67 proliferative index (median 21%, inter quartile range [IQR] 15–30), ER (median 90%, IQR 84–95), PR (median 50%, IQR 15–80) and number of bone metastases (median 5, IQR 3–10). HER2 status was dichotomized into 0 and low (with “low” including HER2 status 1 and 2).

**Table 1:** Clinic-pathological features.

Characteristic	*N* = 1399
**PremenopausalState**	
**No**	1100 (79%)
**Yes**	299 (21%)
**Age**	
**<65**	815 (58%)
**>/=65**	584 (42%)
**PerformanceStatus**	
**ECOG 0**	1195 (85%)
**ECOG 1**	204 (15%)
**Histology**	
**Ductal**	1011 (72%)
**Lobular**	304 (22%)
**Other**	84 (6%)
**Ki67**	
**Median**	21 (15 - 30)
**Grading**	
**G1/G2**	951 (68%)
**G3**	448 (32%)
**ER**	
**Median**	90 (84 -95)
**PR**	
**Median**	50 (15–80)
**HER2**	
**0**	897 (64%)
**1**	332 (24%)
**2**	170 (12%)
**Neo or Adjuvant Chemotherapy**	
**No**	758 (54%)
**Yes**	641 (46%)
**Adjuvant Endocrine Therapy**	
**No**	493 (35%)
**Yes**	906 (65%)
**Bone-only Disease**	
**No**	707 (51%)
**Yes**	692 (49%)
**Bone Metastasis Number**	
**Median**	5 (3–10)
**Visceral Metastasis**	
**No**	761 (54%)
**Yes**	638 (46%)
**Setting**	
**Endocrine Resistant**	599 (43%)
**Endocrine Sensitive**	800 (57%)
**Endocrine Therapy**	
**Aromatase Inhibitor**	942 (67%)
**Fulvestrant**	457 (33%)
**CDK4/6 Inhibitor**	
**Abemaciclib**	219 (16%)
**Palbociclib**	786 (56%)
**Ribociclib**	394 (28%)

Patients treated with palbociclib had poorer ECOG PS (1 *vs* 0), higher ER expression, and visceral metastases at diagnosis; they were more likely to have received neoadjuvant or adjuvant chemotherapy and adjuvant ET, and to have endocrine-resistant disease, compared with patients receiving ribociclib (Supplementary Table 1, available as supplementary data at *The Oncologist* online). Conversely, patients treated with ribociclib were younger, more likely to be pre-menopausal, and to have bone-only disease compared to those treated with palbociclib (Supplementary Table 1, available as supplementary data at *The Oncologist* online). Moreover, patients receiving abemaciclib were more likely to exhibit higher ER expression, endocrine-resistant disease, to have received neoadjuvant or adjuvant chemotherapy and adjuvant ET, and to have a lower number of bone metastases compared to patients receiving ribociclib (Supplementary Table 2, available as supplementary data at *The Oncologist* online). Finally, a higher percentage of patients treated with palbociclib received adjuvant ET compared with those treated with abemaciclib (Supplementary Table 3, available as supplementary data at *The Oncologist* online).

Following propensity score matching (PSM), these baseline differences were mitigated, resulting in successfully matched cohorts (Supplementary Tables 4–6, available as supplementary data at *The Oncologist* online). Similarly, after inverse probability of treatment weighting (IPTW) adjustment, the distributions of clinicopathological characteristics were balanced between the CDK4/6i groups (Supplementary Tables 7–9, available as supplementary data at *The Oncologist* online).

### rwPFS: “unadjusted” cohort

For the three primary pairwise comparisons, statistical significance was assessed using Bonferroni-adjusted p-values (two-sided *α* = 0.0167); other analyses were exploratory.

**Figure 1:**
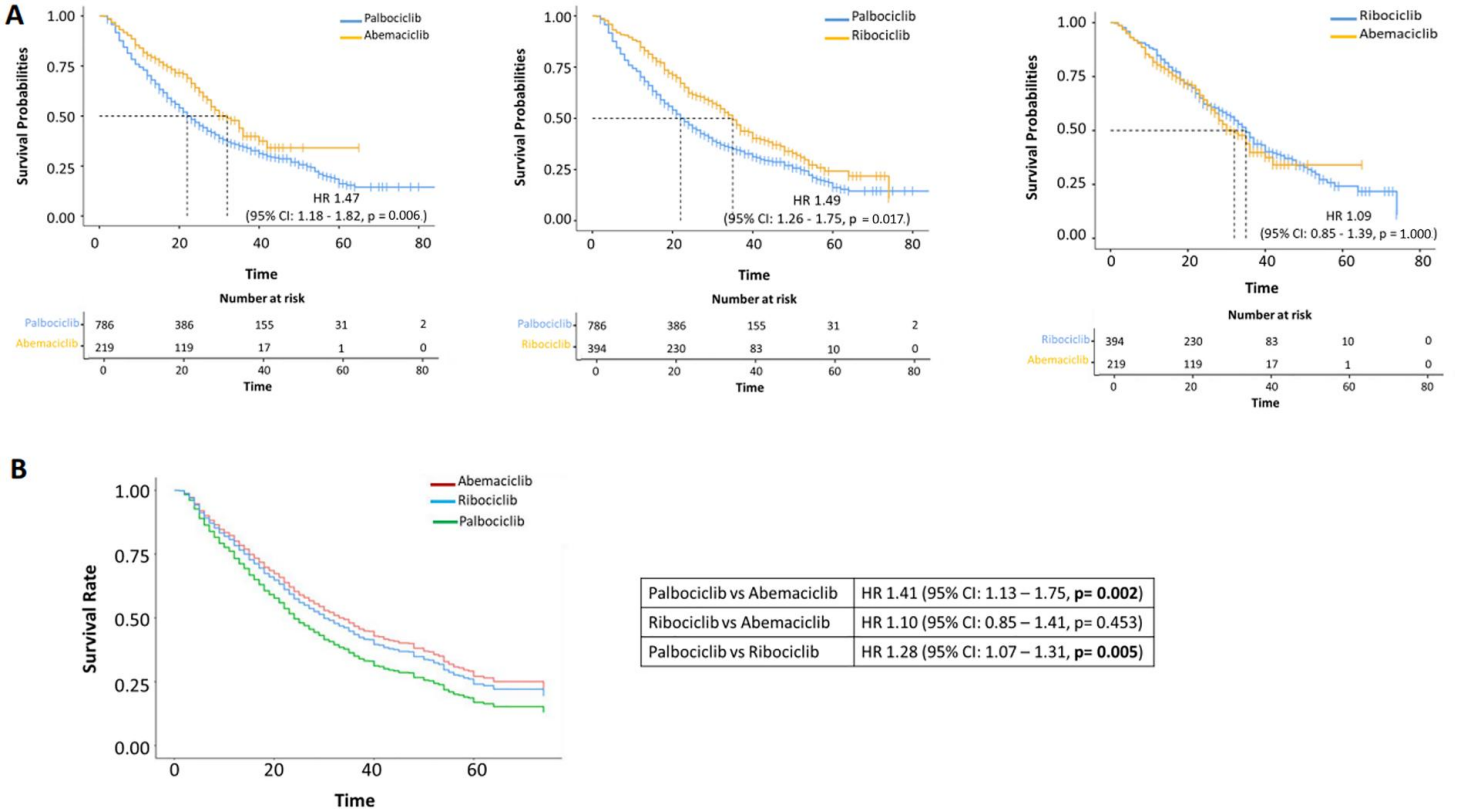
(A) Kaplan–Meier estimates of rwPFS and (B) adjusted Kaplan–Meier curves for rwPFS from the multivariable Cox model in the “unadjusted” cohort; *p*-values for the three primary pairwise comparisons were Bonferroni-adjusted (two-sided *α* = 0.0167)

The median follow-up duration for patients was 39 months (95% CI: 37–40); the median follow-up periods for patients treated with ribociclib, abemaciclib and palbociclib were 34 months (95% CI: 32–39), 28 months (95% CI: 26–31) and 41 months (95% CI: 40–43), respectively. The global median rwPFS was 27.0 months (95% CI: 25.0–30.0). Palbociclib was associated with a shorter rwPFS (median 22 months; 95% CI: 21–25) compared to abemaciclib (median 32 months; 95% CI: 28–36) (HR = 1.47, 95% CI: 1.18–1.82, Bonferroni-adjusted *p = *0.006) (Figure 1A—left panel). Exploratory subgroup analyses showed broadly consistent trends, with palbociclib being generally associated with shorter rwPFS compared with abemaciclib; however, estimates were less precise and did not show clear differences in some strata (including ECOG PS 1, lobular histology, low ER expression, and bone-only disease) (Supplementary Figure 1, available as supplementary data at *The Oncologist* online). Similarly, patients treated with palbociclib had a lower rwPFS compared to those receiving ribociclib (median 35 months; 95% CI: 32–39) (HR = 1.49, 95% CI: 1.26–1.75, Bonferroni-adjusted *p = *0.017) (Figure 1A, central panel).

In exploratory subgroup analyses, the palbociclib–ribociclib comparison showed broadly consistent trends across most subgroups, with inconclusive results in some strata (e.g., ECOG PS 1) likely due to limited sample size and fewer events (Supplementary Figure 2, available as supplementary data at *The Oncologist* online). No significant differences were observed between abemaciclib and ribociclib in all subgroups (Bonferroni-adjusted *p = *1.000) (Figure 1A—right panel; Supplementary Figure 3, available as supplementary data at *The Oncologist* online).

Multivariate Cox regression analysis showed that patients treated with palbociclib had worse rwPFS compared to those treated with abemaciclib (HR = 1.41, 95% CI: 1.13–1.75, *p = *0.002). Additionally, palbociclib was associated with lower rwPFS compared to ribociclib (HR = 1.28, 95% CI: 1.07–1.31, *p = *0.005), independent of clinicopathological features (Table 2; Figure 1B). No significant differences were observed between abemaciclib and ribociclib (*p = *0.453). This analysis also revealed that an ECOG PS of 1 and a higher number of bone metastases were independently associated with shorter rwPFS, whereas bone-only disease was correlated with a longer rwPFS (Table 2). Additionally, an endocrine-resistant setting and low PR expression were associated with a lower rwPFS. (Table 2).

**Table 2:** Multivariate analysis for rwPFS.

rwPFS	All	HR (univariate)	HR (multivariate)
**Premenopausal State**			
**No**	1100 (78.6)	–	–
**Yes**	299 (21.4)	0.90 (0.76–1.07, *p = *0.221)	0.97 (0.81–1.17, *p = *0.756)
**Age**			
**<65**	815 (58.3)	–	–
**>/=65**	584 (41.7)	0.97 (0.84–1.11, *p = *0.646)	0.92 (0.79–1.08, *p = *0.304)
**Performance Status**			
**ECOG 0**	1195 (85.4)	–	–
**ECOG 1**	204 (14.6)	**1.51 (1.26–1.81, *p < *0.001)**	**1.45 (1.20–1.75, *p < *0.001)**
**Histology**			
**Ductal**	1011 (72.3)	–	–
**Lobular**	304 (21.7)	1.10 (0.94–1.30, *p = *0.229)	1.10 (0.93–1.30, *p = *0.280)
**Other**	84 (6.0)	0.97 (0.73–1.29, *p = *0.834)	0.84 (0.63–1.13, *p = *0.247)
**Ki67**			
**High**	699 (50.0)	–	–
**Low**	700 (50.0)	0.92 (0.80–1.05, *p = *0.202)	0.98 (0.85–1.13, *p = *0.788)
**Grading**			
**G1/G2**	951 (68.0)	–	–
**G3**	448 (32.0)	**1.23 (1.06–1.41, *p = *0.005)**	1.13 (0.97–1.31, *p = *0.127)
**ER**			
**Low**	815 (58.3)	1.13 (0.99–1.29, *p = *0.078)	1.07 (0.93–1.23, *p = *0.320)
**High**	584 (41.7)	–	–
**PR**			
**Low**	722 (51.6)	**1.25 (1.09–1.43, *p = *0.001)**	**1.18 (1.02–1.35, *p = *0.023)**
**High**	677 (48.4)	–	–
**HER2**			
**0**	897 (64.1)	–	–
**Low**	502 (35.9)	1.05 (0.91–1.21, *p = *0.520)	1.07 (0.93–1.23, *p = *0.355)
**Neo or Adjuvant Chemotherapy**			
**No**	758 (54.2)	–	–
**Yes**	641 (45.8)	**1.21 (1.06–1.38, *p = *0.006)**	1.07 (0.91–1.27, *p = *0.416)
**Adjuvant Endocrine Therapy**			
**No**	493 (35.2)	–	–
**Yes**	906 (64.8)	**1.29 (1.11–1.49, *p = *0.001)**	1.14 (0.95–1.37, *p = *0.155)
**Bone–only Disease**			
**No**	707 (50.5)	–	–
**Yes**	692 (49.5)	**0.63 (0.55–0.72, *p < *0.001)**	**0.64 (0.46–0.87, *p = *0.005)**
**Bone Metastasis Number**			
**Low**	726 (51.9)	–	–
**High**	692 (49.5)	**1.18 (1.03–1.35, *p = *0.020)**	**1.20 (1.04–1.38, *p = *0.011)**
**Visceral Metastasis**			
**No**	761 (54.4)	–	–
**Yes**	638 (45.6)	**1.51 (1.32–1.73, *p < *0.001)**	1.00 (0.73–1.36, *p = *0.980)
**Setting**			
**Endocrine Resistant**	599 (42.8)	–	–
**Endocrine Sensitive**	800 (57.2)	**0.65 (0.57–0.75, *p < *0.001)**	**0.73 (0.60–0.87, *p = *0.001)**
**Endocrine Therapy**			
**Aromatase Inhibitor**	942 (67.3)	–	–
**Fulvestrant**	457 (32.7)	**1.34 (1.17–1.55, *p < *0.001)**	1.04 (0.86–1.25, *p = *0.703)
**CDK4/6 Inhibitor**			
**Abemaciclib**	219 (15.7)	–	–
**Palbociclib**	786 (56.2)	**1.44 (1.16–1.79, *p = *0.001)**	**1.41 (1.13–1.75, *p = *0.002)**
**Ribociclib**	394 (28.2)	0.97 (0.76–1.23, *p = *0.800)	1.10 (0.86–1.41, *p = *0.453)

*Note:* Bold values indicate statistically significant results (*p* < 0.05).

### rwPFS: “adjusted” cohorts

**Figure 2:**
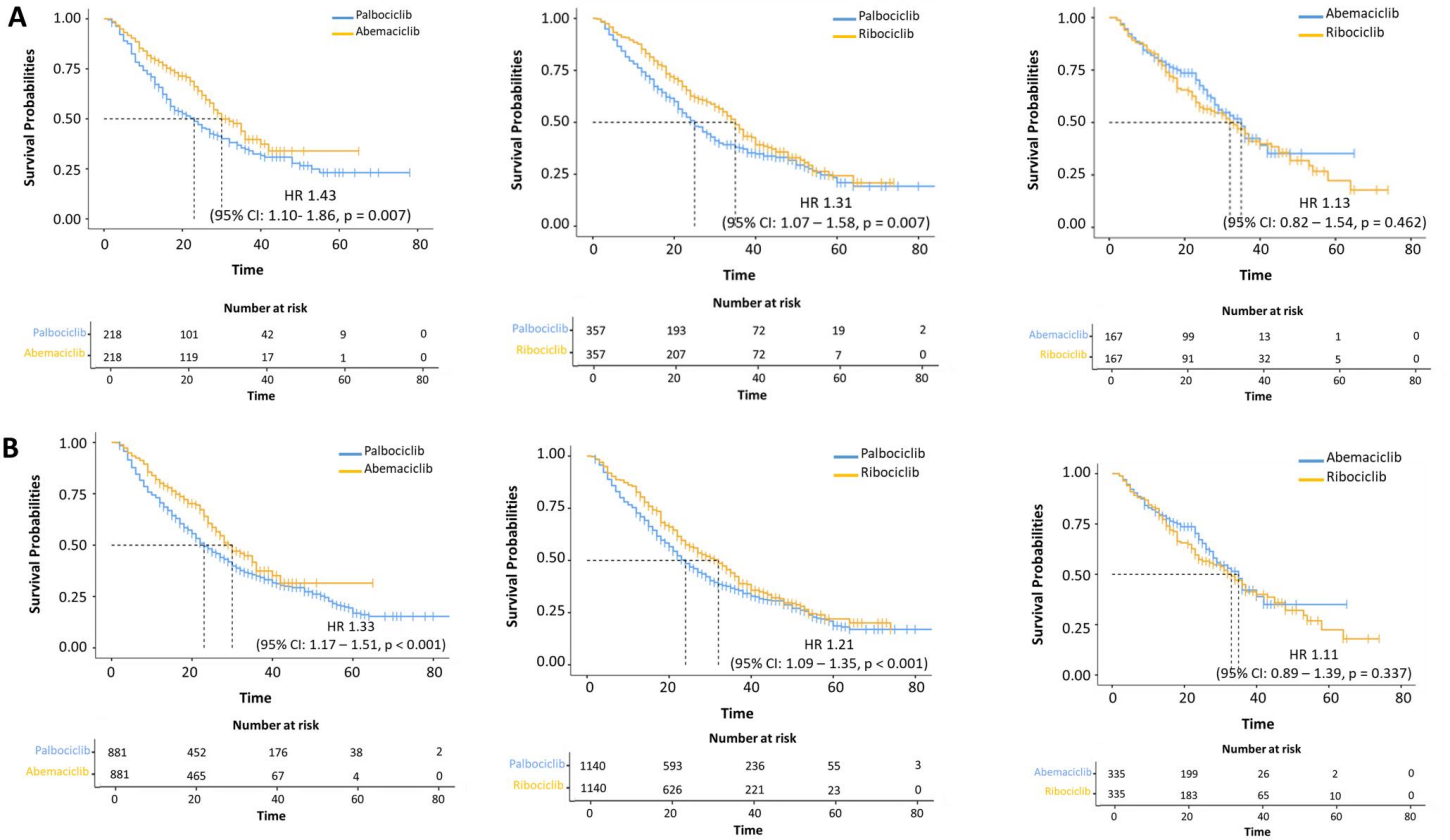
Kaplan-Meier estimates of rwPFS in the “PSM” (A) and “IPTW” (B) cohort.

In the “PSM” cohort, patients treated with palbociclib exhibited a shorter rwPFS (median 23 months; 95% CI: 17–28) compared to those receiving abemaciclib (median 30 months; 95% CI: 28–36) (HR = 1.43, 95% CI: 1.10–1.86, *p = *0.007) (Figure 2A—left panel). Similarly, patients treated with palbociclib experienced a lower rwPFS (median 25 months; 95% CI: 22–29) compared to ribociclib (median 35 months; 95% CI: 32–39) (HR = 1.31, 95% CI: 1.07– 1.58, *p = *0.007) (Figure 2A—central panel). There were no significant differences in rwPFS between the abemaciclib and ribociclib groups (*p = *0.462) (Figure 2A—right panel).

The “IPTW” cohort confirmed these findings, showing that patients treated with palbociclib exhibited a shorter rwPFS (median 23 months; 95% CI: 21–26) compared to those treated with abemaciclib (median 30 months; 95% CI: 28–33) (HR = 1.33, 95% CI: 1.17–1.51, *p < *0.001) (Figure 2B—left panel). Additionally, patients receiving palbociclib had a lower rwPFS (median 24 months; 95% CI: 22–25) compared to those treated with ribociclib (median 32 months; 95% CI: 28–34) (HR = 1.21, 95% CI: 1.09 –1.35, *p < *0.001) (Figure 2B—central panel). Finally, we found no significant differences in rwPFS between patients treated with abemaciclib and ribociclib (*p = *0.337) (Figure 2A—right panel).

### OS exploratory analysis

**Figure 3:**
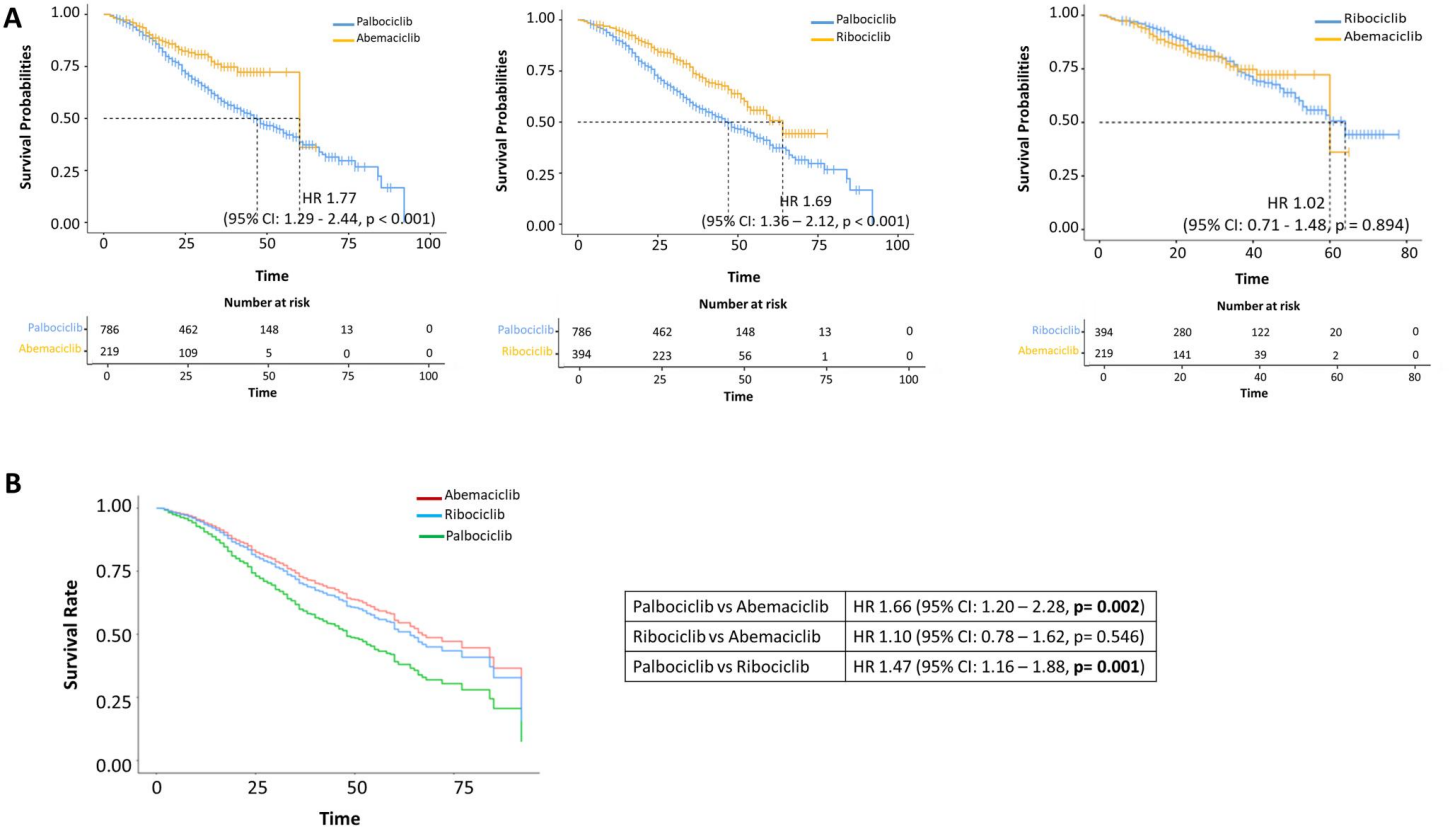
(A) Kaplan-Meier estimates of OS and (B) adjusted Kaplan-Meier Curves for OS from Cox-Multivariate Analysis in the “unadjusted” cohort.

The global median OS was 54.0 months (95% CI: 49.0–60.0). Patients treated with palbociclib exhibited a lower OS (median 47 months; 95% CI: 42–53) than those receiving abemaciclib (median 60 months; 95% CI: 60–not reached) (HR = 1.77, 95% CI: 1.29–2.44, *p < *0.001) (Figure 3A—left panel). Additionally, patients treated with palbociclib had a lower OS compared to those receiving ribociclib (median 64 months; 95% CI: 53–not reached) (HR = 1.69, 95% CI: 1.36–2.12, *p < *0.001) (Figure 3A—central panel). There were no significant differences in OS between the abemaciclib and ribociclib groups (*p = *0.894) (Figure 3A—right panel).

Exploratory OS subgroup analyses showed broadly consistent trends, with palbociclib generally associated with shorter OS versus abemaciclib and ribociclib, but estimates were less precise and should be interpreted cautiously (Supplementary Figures 4–6, available as supplementary data at *The Oncologist* online).

Multivariate Cox regression analysis showed that patients treated with palbociclib had reduced OS compared to those treated with abemaciclib (HR = 1.66, 95% CI: 1.20–2.28, *p = *0.002), and to those treated with ribociclib (HR = 1.47, 95% CI: 1.16–1.88, *p = *0.001). No significant differences between the abemaciclib and ribociclib groups (*p = *0.546). (Table 3; Figure 3B).

**Table 3:** Multivariate analysis for OS.

OS	All	HR (univariate)	HR (multivariate)
**Premenopausal State**			
**No**	1100 (78.6)	–	–
**Yes**	299 (21.4)	0.82 (0.66–1.03, *p = *0.090)	1.01 (0.79–1.30, *p = *0.909)
**Age**			
**<65**	815 (58.3)	–	–
**>/=65**	584 (41.7)	1.11 (0.93–1.32, *p = *0.256)	0.98 (0.80–1.19, *p = *0.826)
**Performance Status**			
**ECOG 0**	1195 (85.4)	–	–
**ECOG 1**	204 (14.6)	**2.33 (1.89–2.88, *p < *0.001)**	**2.09 (1.68–2.62, *p < *0.001)**
**Histology**			
**Ductal**	1011 (72.3)	–	–
**Lobular**	304 (21.7)	1.20 (0.97–1.47, *p = *0.090)	1.12 (0.90–1.39, *p = *0.312)
**Other**	84 (6.0)	1.04 (0.71–1.50, *p = *0.853)	0.89 (0.61–1.30, *p = *0.535)
**Ki67**			
**High**	699 (50.0)	–	–
**Low**	700 (50.0)	0.87 (0.73–1.04, *p = *0.133)	0.92 (0.77–1.11, *p = *0.399)
**Grading**			
**G1/G2**	951 (68.0)	–	–
**G3**	448 (32.0)	1.14 (0.95–1.37, *p = *0.170)	1.06 (0.87–1.29, *p = *0.584)
**ER**			
**Low**	815 (58.3)	**1.19 (1.00–1.42), *p = *0.046)**	1.15 (0.95–1.37, *p = *0.148)
**High**	584 (41.7)	–	–
**PR**			
**Low**	722 (51.6)	**1.21 (1.02–1.45, *p = *0.032)**	1.17 (0.98–1.40, *p = *0.092)
**High**	677 (48.4)	–	–
**HER2**			
**0**	897 (64.1)	–	–
**Low**	502 (35.9)	0.91 (0.75–1.09, *p = *0.295)	0.89 (0.73–1.07, *p = *0.210)
**Neo or Adjuvant Chemotherapy**			
**No**	758 (54.2)	–	–
**Yes**	641 (45.8)	0.92 (0.77–1.10, *p = *0.375)	0.89 (0.72–1.10, *p = *0.284)
**Adjuvant Endocrine Therapy**			
**No**	493 (35.2)	–	–
**Yes**	906 (64.8)	1.06 (0.88–1.28, *p = *0.525)	1.09 (0.87–1.37, *p = *0.463)
**Bone-only Disease**			
**No**	707 (50.5)	–	–
**Yes**	692 (49.5)	**0.58 (0.49–0.70, *p < *0.001)**	**0.49 (0.34–0.72, *p < *0.001)**
**Bone Metastasis Number**			
**Low**	726 (51.9)	–	–
**High**	673 (48.1)	**1.26 (1.06–1.50, *p = *0.010)**	**1.26 (1.05–1.51, *p = *0.012)**
**Visceral Metastasis**			
**No**	761 (54.4)	–	–
**Yes**	638 (45.6)	**1.49 (1.25–1.78, *p < *0.001)**	0.79 (0.54–1.14, *p = *0.206)
**Setting**			
**Endocrine Resistant**	599 (42.8)	–	–
**Endocrine Sensitive**	800 (57.2)	**0.76 (0.63–0.90, *p = *0.002)**	0.80 (0.63–1.02, *p = *0.078)
**EndocrineTherapy**			
**Aromatase Inhibitor**	942 (67.3)	–	–
**Fulvestrant**	457 (32.7)	**1.20 (1.00–1.44, *p = *0.049)**	1.00 (0.78–1.27, *p = *0.976)
**CDK4/6 Inhibitor**			
**Abemaciclib**	219 (15.7)	–	–
**Palbociclib**	786 (56.2)	**1.74 (1.27–2.39, *p = *0.001)**	**1.66 (1.20–2.28, *p = *0.002)**
**Ribociclib**	394 (28.2)	1.02 (0.71–1.46, *p = *0.916)	1.12 (0.78–1.62, *p = *0.546)

*Note:* Bold values indicate statistically significant results (*p* < 0.05).

**Figure 4:**
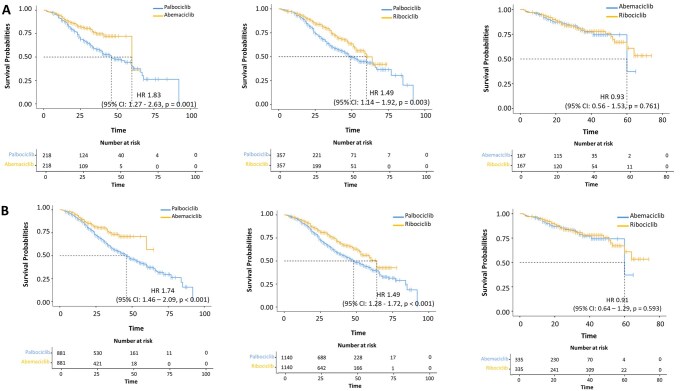
Kaplan-Meier estimates of OS in the “PSM” (A) and “IPTW” (B) cohort.

Palbociclib was correlated with worse OS (median 46 months; 95% CI: 42–52) compared to abemaciclib (median not reached; 95% CI: 60–not reached) (HR = 1.83, 95% CI: 1.27–2.63, *p < *0.001), even after “PSM” adjustment (Figure 4A—left panel). Similarly, in the “IPTW”, palbociclib was associated with reduced OS (median 46 months; 95% CI: 42–52) compared to abemaciclib (median not reached; 95% CI: 60–not reached) (HR = 1.74, 95% CI: 1.46–2.09, *p < *0.001) (Figure 4B—left panel). Additionally, palbociclib was associated with a worse OS (median 49 months; 95% CI: 44–66) compared to ribociclib (median 60 months; 95% CI: 53–not reached) (HR = 1.49, 95% CI: 1.14–1.92, *p = *0.003) (Figure 4 A—central panel) in the “PSM” cohort. Likewise, palbociclib was correlated with a worse OS (median 48 months; 95% CI: 45–53) compared to ribociclib median 64 months; 95% CI: 59–not reached) (HR = 1.49, 95% CI: 1.28–1.72, *p < *0.001), after “IPTW” adjustment (Figure 4B—central panel). Finally, no significant differences were observed between patients treated with abemaciclib and ribociclib in both the IPTW (*p = *0.761) and the PSM cohort (*p = *0.593) (Figure 4A—right panel).

### Sensitivity analysis

The results of the leave-one-out sensitivity analysis are presented in Supplementary Tables 10–11, available as supplementary data at *The Oncologist* online. Unless otherwise specified, *p*-values reported for sensitivity analyses are nominal and intended for consistency assessment. Across these sensitivity analyses, the direction of the associations was generally consistent with the main analysis, and exclusion of any single center did not materially change the overall estimates.

To further assess the robustness of our findings against unmeasured confounding, we calculated the E-values for the observed HRs and their confidence intervals. E-values suggested that a relatively strong unmeasured confounder would be required to fully explain away the observed associations: 2.12 (rwPFS) and 2.30 (OS) for palbociclib versus abemaciclib, and 1.92 (rwPFS) and 2.17 (OS) for palbociclib versus ribociclib; however, residual confounding cannot be excluded.

In additional sensitivity analyses stratified by treatment line (year-adjusted Cox models), palbociclib was associated with shorter rwPFS versus abemaciclib (first line: HR 1.53, 95% CI 1.17–2.00; *p = *0.002; second line: HR 1.56, 95% CI 1.01–2.41; *p = *0.046) and versus ribociclib in first line (HR 1.31, 95% CI 1.08–1.60; *p = *0.007); restricting the analysis to patients initiating therapy in 2020 or later (adjusted for line and year), palbociclib remained associated with shorter rwPFS versus abemaciclib (HR 1.40, 95% CI 1.04–1.88; *p = *0.025). Similarly, in first line palbociclib was associated with worse OS versus abemaciclib (HR 1.72, 95% CI 1.19–2.49; *p = *0.004) and versus ribociclib (HR 1.63, 95% CI 1.25–2.11; *p < *0.001), whereas in second line no statistically significant OS differences were observed; in the post-2020 analysis (adjusted for line and year), palbociclib remained associated with worse OS versus abemaciclib (HR 1.69, 95% CI 1.11–2.58; *p = *0.014) (Supplementary Table 12, available as supplementary data at *The Oncologist* online).

## Discussion

The study evaluated the rwPFS of 1399 HR+/HER2- mBC patients with bone metastases treated with ET plus CDK4/6i as first- or second-line therapy. The findings revealed significant differences in rwPFS among the three treatments: patients treated with palbociclib exhibited shorter rwPFS compared to those receiving abemaciclib and ribociclib. These differences persisted even after adjusting for baseline characteristics using PSM and IPTW. The multivariate Cox regression analysis showed that palbociclib was associated with worse rwPFS compared to abemaciclib and ribociclib, independent of clinicopathological features. Specifically, the median rwPFS for palbociclib was 22 months, whereas it was 32 months for abemaciclib and 35 months for ribociclib. Similarly, explorative OS analysis showed a reduced median OS for palbociclib (47 months), compared to abemaciclib (60 months) and ribociclib (64 months), even after adjusting using PSM and IPTW, and multivariate Cox regression analysis.

The study’s data indicated slightly better outcomes compared to the registration trials of palbociclib (PALOMA 2 and 3),^14–16^ abemaciclib (MONARCH 2 and 3),^9–11^ and ribociclib (MONALEESA 2 and 3).^12–13^ For instance, in the PALOMA-2 trial, the median PFS for palbociclib plus letrozole was 24.8 months, whereas our study observed a median rwPFS of 37 months in endocrine-sensitive patients treated with palbociclib. The PALOMA-3 trial reported a median PFS and OS of 9.5 and 34.8 months, respectively, for patients treated with palbociclib plus fulvestrant. In contrast, our study showed a median rwPFS and OS of 18 and 43 months, respectively, in endocrine-resistant patients treated with palbociclib. For abemaciclib, the MONARCH-2 trial reported a median PFS and OS of 16.4 and 46.7 months, respectively, while our study’s mean rwPFS and OS were 27 and 59.7 months in endocrine-resistant patients. Similarly, the MONARCH-3 trial showed a median PFS and OS of 28 and 66.8 months, compared to the 36 months rwPFS and OS not reached in our endocrine-sensitive patients. Regarding ribociclib, the MONALEESA-2 trial reported a median PFS of 25.3 months, which is lower than the 36 months observed in our cohort of endocrine sensitive patients treated with ribociclib. Finally, the MONALEESA-3 trial’s median PFS was 20.5 months, compared to our study’s mean rwPFS of 27 months.

The overall better prognosis observed in the study might be attributed, at least in part, to a lower percentage of patients with ECOG PS 1 and a higher percentage of patients with bone-only disease, which is known to be associated with favorable outcomes. For example, in the PALOMA-2 trial, 40% of patients had an ECOG PS of 1, compared to only 19% of endocrine-sensitive patients treated with palbociclib in our study. Moreover, bone-only disease was present in 23% of patients in the PALOMA-2 trial, compared to 48% in our cohort of endocrine-sensitive patients treated with palbociclib. Similarly, in the PALOMA-3 trial, 41% of patients had an ECOG PS of 1, compared to 16% in our cohort of endocrine-resistant patients treated with palbociclib. Additionally, 21% of patients in the PALOMA-3 trial had bone-only disease, compared to 45% in our study.

In the MONARCH-2 trial, 31% of patients had an ECOG PS of 1, compared to 14% in our cohort of endocrine-resistant patients treated with abemaciclib, while 28% had bone-only disease, compared to 54% in our study. Similarly, in the MONARCH-3 trial, 41% of patients had an ECOG PS of 1, compared to 17% of our endocrine-sensitive patients treated with abemaciclib, while 21% had bone-only disease, compared to 54% in our study.

In the MONALEESA-2 trial, 39% of patients had an ECOG PS of 1, compared to 9% in our cohort of endocrine-sensitive patients treated with ribociclib, while 21% had bone-only disease, compared to 53% in our study. Similarly, in the MONALEESA-3 trial, 35% of patients had an ECOG PS of 1, compared to 6% in our cohort of endocrine-resistant patients treated with ribociclib, while 21% had bone-only disease, compared to 52% in our study.

Furthermore, many factors can significantly influence rwPFS and OS, including comorbidities, subsequent therapies, and differing interpretations of radiological images. More frequent and thorough clinical and radiological assessments in pivotal trials might explain inferior outcomes. In fact, in trials, tumour assessments occurred every eight weeks, while in clinical practice, they are often conducted less frequently, sometimes every 12 weeks. Moreover, disease progression in trials relied on standardized interpretation of CT or MRI, whereas in real-world settings, radiologists’ and oncologists’ assessments play a key role. For this reason, caution is required when comparing real-world data with randomized controlled trials.

Exploratory subgroup analyses showed broadly similar trends across most strata but should be interpreted cautiously given limited power for interaction testing and multiple comparisons. However, in the ECOG PS 1 subgroup, the rwPFS and OS of patients treated with palbociclib were like those treated with abemaciclib or ribociclib, a finding that may be clinically relevant in routine practice, where tolerability considerations can influence treatment choice.

Our study’s findings are consistent with those of the PALMARES-2 study, which also assessed these treatments in a real-world setting and demonstrated the superiority of abemaciclib and ribociclib over palbociclib in terms of rwPFS.^25^ This study reported a global median rwPFS of 34.9 months, which is slightly higher than the 27 months observed in our study. This difference is likely due, at least in part, to the higher percentage of patients treated with palbociclib in our cohort (56% *vs* 40%) and maybe because all patients in our cohort had metastatic disease. Other real-world studies have compared the efficacy of the three CDK4/6i with similar results.^26–28^ A monocentric study reported that ribociclib and abemaciclib showed superior PFS and OS compared to palbociclib, in HR+ mBC patients.^26^ Similarly, the study by Gehrchen et al. on the real-world effectiveness of CDK 4/6i in the same setting found that abemaciclib and ribociclib had prolonged PFS compared to palbociclib.^27^ Another study by Cejuela et al. directly compared abemaciclib, palbociclib, and ribociclib in real-world data and it showed that abemaciclib was associated with a significant benefit in terms of PFS in endocrine-resistant patients and those without visceral involvement.^28^ A recent large real-world EHR-based study by Rugo et al.^29^ reported no statistically significant differences in OS among patients with HR+/HER2− mBC treated with palbociclib, ribociclib, or abemaciclib. Importantly, rwPFS and subgroup results from these data have also been presented at ESMO 2025 and SABCS 2025 and should be interpreted alongside our findings. Differences between those analyses and the present study may reflect heterogeneity in study design and case-mix, including our focus on patients with bone metastases at metastatic diagnosis, the inclusion of later-line CDK4/6i initiation, potential calendar-time and drug-adoption effects, variability in endocrine therapy backbones, and differences in endpoint ascertainment and censoring typical of retrospective real-world datasets. Accordingly, our results should be viewed as hypothesis-generating and applicable primarily to this specific bone-metastatic population rather than as definitive evidence of causal superiority among CDK4/6 inhibitors. Two network meta-analysis highlighted also no significant differences in survival among the different CDK4/6i.^30–31^ A unique feature of this study is that all patients enrolled had bone metastases at diagnosis of metastatic disease. Bone metastases present a distinct challenge in breast cancer treatment, as they not only lead to SRE but they also modify the tumor microenvironment and can modify drug efficacy. This altered microenvironment can facilitate tumor growth and induce resistance to treatments, making patients with bone metastases particularly difficult to manage effectively. Additionally, the presence of bone metastases could influence the varying efficacy of CDK4/6i. Our previous work demonstrated that palbociclib exhibited a lower anti-tumor effect compared to ribociclib and abemaciclib in preclinical models of BC bone metastases.^20^ These differences could be attributed to the distinct selectivity profiles of the three compounds. Biochemical interaction analyses revealed that while all drugs are highly selective for CDK4/6, abemaciclib also binds to multiple other kinases.^32^ Additionally, palbociclib has similar potency against cyclin D1/CDK4 and cyclin D2/CDK6,^33^ whereas abemaciclib and ribociclib show greater potency against CDK4 than CDK6.^34^ The varying spectrum and degree of interactions of these agents could influence their clinical performance.

Other factors may have influenced our results, such as the different periods during which the three CDKis were introduced into clinical practice. Indeed, the lower experience of clinicians with palbociclib, being the first CDK 4/6i approved, could have influenced its efficacy in real-world settings. As ribociclib and abemaciclib were approved later, clinicians had more experience and knowledge about the class of CDK4/6i by the time these drugs became available, potentially leading to better management and outcomes. Although this study features a large sample size and employs robust statistical methods to adjust for measured confounding factors, several limitations must be considered. First, and most importantly, the retrospective, non-randomized design resulted in clinically meaningful baseline imbalances across treatment groups, raising concern for confounding by indication (channeling bias), with palbociclib appearing more frequently prescribed to patients with poorer prognostic characteristics and/or in later-line settings. Although we applied multivariable adjustment and propensity-based approaches, residual and unmeasured confounding cannot be excluded, and therefore the observed differences should be interpreted as associative rather than causal. To further address this issue, we performed additional sensitivity analyses stratified by line of therapy (first-line-only and second-line-only models). Line-stratified analyses showed broadly consistent trends but reduced precision, especially in second line, highlighting the potential impact of treatment-line distribution and residual confounding on the overall comparisons.

Post-progression (subsequent-line) treatment patterns could not be robustly compared due to incomplete and non-uniform capture across centers, which may contribute to residual confounding.

Moreover, although the follow-up period is considerable, it may not be sufficient to capture long-term outcomes for all patients. Finally, while the use of real-world data is valuable, it carries inherent limitations, including incomplete data capture, variability in data quality, and missing information.

In conclusion, the results of this study are consistent with previous findings suggesting that palbociclib may be less effective than other CDK 4/6i in patients with bone metastases. However, it remains unclear whether a definitive ranking of the three drugs can be established. Future prospective studies are warranted to validate these results and explore the underlying mechanisms.

## Supplementary Material

oyag146_Supplementary_Data

## Data Availability

All data generated or analyzed during this study are included in the article and its supplementary materials.

## References

[1] Sung H , Ferlay J, Siegel RL, et al Global cancer statistics 2020: GLOBOCAN estimates of incidence and mortality worldwide for 36 cancers in 185 countries. CA Cancer J Clin. 2021;71:209-249. 33538338 10.3322/caac.21660

[2] Wolff AC , Somerfield MR, Dowsett M, et al Human epidermal growth factor receptor 2 testing in breast cancer: ASCO-College of American pathologists guideline update. J Clin Oncol. 2023;41:3867-3872. 10.1200/JCO.22.02864 37284804

[3] Wang X , Zhao S, Xin Q, et al Recent progress of CDK4/6 inhibitors’ current practice in breast cancer. Cancer Gene Ther. 2024;31:1283-1291. 10.1038/s41417-024-00747-x 38409585 PMC11405274

[4] Goel S , DeCristo MJ, McAllister SS, Zhao JJ. CDK4/6 inhibition in cancer: beyond cell cycle arrest. Trends Cell Biol. 2018;28:911-925. 10.1016/j.tcb.2018.07.002 30061045 PMC6689321

[5] Goel S , Bergholz JS, Zhao JJ. Targeting CDK4 and CDK6 in cancer. Nat Rev Cancer. 2022;22:356-372. 10.1038/s41568-022-00456-3 35304604 PMC9149100

[6] Ding L , Cao J, Lin W, et al The roles of cyclin-dependent kinases in cell-cycle progression and therapeutic strategies in human breast cancer. Int J Mol Sci. 2020;21:1960. 10.3390/ijms21061960 32183020 PMC7139603

[7] Huang J , Zheng L, Sun Z, Li J. CDK4/6 inhibitor resistance mechanisms and treatment strategies (review). Int J Mol Med. 2022;50:128. 10.3892/ijmm.2022.5184 36043521 PMC9448295

[8] Watt AC , Goel S. Cellular mechanisms underlying response and resistance to CDK4/6 inhibitors in the treatment of hormone receptor-positive breast cancer. Breast Cancer Res. 2022;24:17. 10.1186/s13058-022-01510-6 35248122 PMC8898415

[9] Goetz MP , Toi M, Huober J, et al MONARCH 3: abemaciclib as initial therapy for advanced breast cancer. J Clin Oncol. 2017;35:3638-3646. 10.1200/JCO.2017.75.6155 28968163

[10] Sledge GW Jr , Toi M, Neven P, et al MONARCH 2: abemaciclib in combination with fulvestrant in women with HR+/HER2- Advanced breast cancer who had progressed while receiving endocrine therapy. J Clin Oncol. 2017;35:2875-2884. 10.1200/JCO.2017.73.7585 28580882

[11] Sledge GW , Toi M, Neven P, et al The effect of abemaciclib plus fulvestrant on overall survival in hormone receptor-positive, ERBB2-negative breast cancer that progressed on endocrine therapy - MONARCH 2: a randomized clinical trial. JAMA Oncol. 2020;6:116-124. 10.1001/jamaoncol.2019.4782 31563959 PMC6777264

[12] Hortobagyi GN , Stemmer SM, Burris HA, et al Overall survival with ribociclib plus letrozole in advanced breast cancer. N Engl J Med. 2022;386:942-950. 10.1056/NEJMoa2114663 35263519

[13] Slamon DJ , Neven P, Chia S, et al Overall survival with ribociclib plus fulvestrant in advanced breast cancer. N Engl J Med. 2020;382:514-524. 10.1056/NEJMoa1911149 31826360

[14] Finn RS , Martin M, Rugo HS, et al Palbociclib and letrozole in advanced breast cancer. N Engl J Med. 2016;375:1925-1936. 10.1056/NEJMoa1607303 27959613

[15] Turner NC , Slamon DJ, Ro J, et al Overall survival with palbociclib and fulvestrant in advanced breast cancer. N Engl J Med. 2018;379:1926-1936. 10.1056/NEJMoa1810527 30345905

[16] Rugo HS , Finn RS, Dieras V, et al Palbociclib plus letrozole as first-line therapy in estrogen receptor-positive/human epidermal growth factor receptor 2-negative advanced breast cancer with extended follow-up. Breast Cancer Res Treat. 2019;174:719-729. 10.1007/s10549-018-05125-4 30632023 PMC6438948

[17] Shao H , Varamini P. Breast cancer bone metastasis: a narrative review of emerging targeted drug delivery systems. Cells. 2022;11:388. 10.3390/cells11030388 35159207 PMC8833898

[18] Pang L , Gan C, Xu J, et al Bone metastasis of breast cancer: Molecular mechanisms and therapeutic strategies. Cancers (Basel). 2022;14:5727. 10.3390/cancers14235727 36497209 PMC9738274

[19] Coleman RE. Clinical features of metastatic bone disease and risk of skeletal morbidity. Clin Cancer Res. 2006;12:6243s-6249s. 10.1158/1078-0432.CCR-06-0931 17062708

[20] Iuliani M , Simonetti S, Ribelli G, et al Biological effects of cyclin-dependent kinase inhibitors ribociclib, palbociclib and abemaciclib on breast cancer bone microenvironment. Int J Mol Sci. 2022;23:2477. 10.3390/ijms23052477 35269621 PMC8910497

[21] Cardoso F , Senkus E, Costa A, et al 4th ESO-ESMO international consensus guidelines for advanced breast cancer (ABC4). Ann Oncol. 2018;29:1634-1657. 10.1093/annonc/mdy192 30032243 PMC7360146

[22] VanderWeele TJ , Ding P. Sensitivity analysis in observational research: introducing the E-value. Ann Intern Med. 2017;167:268-274. 10.7326/M16-2607 28693043

[23] Ho D , Imai K, King G, Stuart EA. MatchIt: nonparametric preprocessing for parametric causal inference. J Stat Softw. 2011;42:1-28. 10.18637/jss.v042.i08

[24] The jamovi project. jamovi (Version 2.5) [Computer Software]. 2024. https://www.jamovi.org

[25] Vernieri C , Provenzano L, Giuliano M, et al Comparison of antitumor efficacy of first-line palbociclib, ribociclib, or abemaciclib in patients with HR+/HER2- aBC: results of the multicenter, real-world, italian study PALMARES-2. J Clin Oncol. 2024;42(suppl 1):Abstract 1234.

[26] Dedic Plavetic N , Čular K, Gudelj D, et al Real-world comparison of the efficacy of three CDK4/6 inhibitors (CDK4/6i) in the first-line treatment of endocrine-sensitive advanced breast cancer (aBC): single institution experience. J Clin Oncol. 2024;42(suppl 1):Abstract 5678.

[27] Gehrchen ML , Berg T, Garly R, et al Real-world effectiveness of CDK 4/6 inhibitors in estrogen-positive metastatic breast cancer. BJC Rep. 2024;2:44-134. 10.1038/s44276-024-00070-w 39516670 PMC11523969

[28] Cejuela M , Gil-Torralvo A, Castilla MÁ, et al Abemaciclib, palbociclib, and ribociclib in real-world data: a direct comparison of first-line treatment for endocrine-receptor-positive metastatic breast cancer. Int J Mol Sci. 2023;24:8488. 10.3390/ijms24108488 37239834 PMC10217927

[29] Rugo HS , Layman RM, Lynce F, et al Comparative overall survival of CDK4/6 inhibitors plus an aromatase inhibitor in HR+/HER2- metastatic breast cancer in the US real-world setting. ESMO Open. 2025;10:104103. 10.1016/j.esmoop.2024.104103 39754979 PMC11758200

[30] Kappel C , Elliott MJ, Kumar V, et al Comparative overall survival of CDK4/6 inhibitors in combination with endocrine therapy in advanced breast cancer. Sci Rep. 2024;14:3129. 10.1038/s41598-024-53151-8 38326452 PMC10850180

[31] Tong F , Lu Y, Ma HF, Shen J. Comparative efficacy and safety of CDK4/6 inhibitors combined with endocrine therapies for HR+/HER2- breast cancer: Systematic review and network meta-analysis. Heliyon. 2024;10:e31583. 10.1016/j.heliyon.2024.e31583 38832268 PMC11145204

[32] Chen P , Lee NV, Hu W, et al Spectrum and degree of CDK drug interactions predicts clinical performance. Mol Cancer Ther. 2016;15:2273-2281. 10.1158/1535-7163.MCT-16-0143 27496135

[33] Fry DW , Harvey PJ, Keller PR, et al Specific inhibition of cyclin-dependent kinase 4/6 by PD 0332991 and associated antitumor activity in human tumor xenografts. Mol Cancer Ther. 2004;3:1427-1438. 15542782

[34] Gelbert LM , Cai S, Lin X, et al Preclinical characterization of the CDK4/6 inhibitor LY2835219: In-vivo cell cycle-dependent/independent anti-tumor activities alone/in combination with gemcitabine. Invest New Drugs. 2014;32:825-837. 10.1007/s10637-014-0125-5 24919854 PMC4169866

