# Supplementary material for: Comparative real-world progression free survival of CDK4/6 inhibitors in HR+/HER2− breast cancer patients with bone metastases
Source: Oncologist. 2026 Apr 16;31(5):oyag146. doi: 10.1093/oncolo/oyag146 (PMC13127761; doi:10.1093/oncolo/oyag146)
Supplement: oyag146_Supplementary_Data [file oyag146_supplementary_data.zip › Supplementary Tables revised.docx]

**Supplementary Table 1:** Clinic-pathological features of patients treated with Palbociclib or Ribociclib

| **Characteristics** | **Palbociclib N = 786 (67%)** | **Ribociclib N = 394 (33%)** | **p-value** |
| --- | --- | --- | --- |
| **Premenopausal State** | 137 (17%) | 116 (29%) | ***<0.001*** |
| **Age** |  |  | ***<0.001*** |
| <65 | 423 (54%) | 267 (68%) |  |
| >/=65 | 363 (46%) | 127 (32%) |  |
| **Performance Status** |  |  | ***<0.001*** |
| ECOG 0 | 650 (83%) | 360 (91%) |  |
| ECOG 1 | 136 (17%) | 34 (8.6%) |  |
| **Histology** |  |  | 0.22 |
| Ductal | 557 (71%) | 294 (75%) |  |
| Lobular | 175 (22%) | 82 (21%) |  |
| Other | 54 (6.9%) | 18 (4.6%) |  |
| **Ki67** |  |  | 0.48 |
| High | 390 (50%) | 204 (52%) |  |
| Low | 396 (50%) | 190 (48%) |  |
| **Grading** |  |  | 0.94 |
| G1/G2 | 533 (68%) | 268 (68%) |  |
| G3 | 253 (32%) | 126 (32%) |  |
| **ER** |  |  | ***0.012*** |
| Low | 443 (56%) | 252 (64%) |  |
| High | 343 (44%) | 142 (36%) |  |
| **PR** |  |  | 0.19 |
| Low | 413 (53%) | 191 (48%) |  |
| High | 373 (47%) | 203 (52%) |  |
| **HER2** |  |  | 0.71 |
| 0 | 506 (64%) | 258 (65%) |  |
| Low | 280 (36%) | 136 (35%) |  |
| **Neo or Adjuvant Chemotherapy** | 379 (48%) | 147 (37%) | ***<0.001*** |
| **Adjuvant Endocrine Therapy** | 537 (68%) | 203 (52%) | ***<0.001*** |
| **Bone-only Disease** | 366 (47%) | 208 (53%) | ***0.044*** |
| **Bone Metastasis Number** |  |  | 0.28 |
| Low | 409 (52%) | 192 (49%) |  |
| High | 377 (48%) | 202 (51%) |  |
| **Visceral Metastasis** | 380 (48%) | 161 (41%) | ***0.015*** |
| **Setting** |  |  | ***<0.001*** |
| Endocrine Resistant | 398 (51%) | 96 (24%) |  |
| Endocrine Sensitive | 388 (49%) | 298 (76%) |  |
| **Endocrine Therapy** |  |  | 0.7 |
| Aromatase Inhibitor | 526 (67%) | 268 (68%) |  |
| Fulvestrant | 260 (33%) | 126 (32%) |  |

**Supplementary Table 2:** Clinic-pathological features of patients treated with Ribociclib or Abemaciclib

| **Characteristic** | **Ribociclib N = 394 (64%)** | **Abemaciclib N = 219 (36%)** | **p-value** |
| --- | --- | --- | --- |
| **Premenopausal State** | 116 (29%) | 46 (21%) | ***0.023*** |
| **Age** |  |  | ***0.008*** |
| <65 | 267 (68%) | 125 (57%) |  |
| >/=65 | 127 (32%) | 94 (43%) |  |
| **Performance Status** |  |  | ***0.009*** |
| ECOG 0 | 360 (91%) | 185 (84%) |  |
| ECOG 1 | 34 (8.6%) | 34 (16%) |  |
| **Histology** |  |  | 0.86 |
| Ductal | 294 (75%) | 160 (73%) |  |
| Lobular | 82 (21%) | 47 (21%) |  |
| Other | 18 (4.6%) | 12 (5.5%) |  |
| **Ki67** |  |  | 0.36 |
| High | 204 (52%) | 105 (48%) |  |
| Low | 190 (48%) | 114 (52%) |  |
| **Grading** |  |  | 0.9 |
| G1/G2 | 268 (68%) | 150 (68%) |  |
| G3 | 126 (32%) | 69 (32%) |  |
| **ER** |  |  | ***0.026*** |
| Low | 252 (64%) | 120 (55%) |  |
| High | 142 (36%) | 99 (45%) |  |
| **PR** |  |  | 0.2 |
| Low | 191 (48%) | 118 (54%) |  |
| High | 203 (52%) | 101 (46%) |  |
| **HER2** |  |  | 0.24 |
| 0 | 258 (65%) | 133 (61%) |  |
| Low | 136 (35%) | 86 (39%) |  |
| **Neo or Adjuvant Chemotherapy** | 147 (37%) | 115 (53%) | ***<0.001*** |
| **Adjuvant Endocrine Therapy** | 203 (52%) | 166 (76%) | ***<0.001*** |
| **Bone Only Disease** | 208 (53%) | 118 (54%) | 0.8 |
| **Bone Metastasis Number** |  |  | ***0.048*** |
| Low | 192 (49%) | 125 (57%) |  |
| High | 202 (51%) | 94 (43%) |  |
| **Visceral Metastasis** | 161 (41%) | 97 (44%) | 0.41 |
| **Setting** |  |  | ***<0.001*** |
| Endocrine Resistant | 96 (24%) | 105 (48%) |  |
| Endocrine Sensitive | 298 (76%) | 114 (52%) |  |
| **Endocrine Therapy** |  |  | 0.91 |
| Aromatase Inhibitor | 268 (32%) | 148 (68%) |  |
| Fulvestrant | 126 (32%) | 71 (32%) |  |

**Supplementary Table 3:** Clinic-pathological features of patients treated with Palbociclib or Abemaciclib

| **Characteristics** | **Palbociclib N = 786 (78%)** | **Abemaciclib N = 219 (22%)** | **p-value** |
| --- | --- | --- | --- |
| **Premenopausal State** | 137 (17%) | 46 (21%) | 0.23 |
| **Age** |  |  | 0.39 |
| <65 | 423 (54%) | 125 (57%) |  |
| >/=65 | 363 (46%) | 94 (43%) |  |
| **Performance Status** |  |  | 0.53 |
| ECOG 0 | 650 (83%) | 185 (84%) |  |
| ECOG 1 | 136 (17%) | 34 (16%) |  |
| **Histology** |  |  | 0.72 |
| Ductal | 557 (71%) | 160 (73%) |  |
| Lobular | 175 (22%) | 47 (21%) |  |
| Other | 54 (6.9%) | 12 (5.5%) |  |
| **Ki67** |  |  | 0.66 |
| High | 390 (50%) | 105 (48%) |  |
| Low | 396 (50%) | 114 (52%) |  |
| **Grading** |  |  | 0.85 |
| G1/G2 | 533 (68%) | 150 (68%) |  |
| G3 | 253 (32%) | 69 (32%) |  |
| **ER** |  |  | 0.68 |
| Low | 443 (56%) | 120 (55%) |  |
| High | 343 (44%) | 99 (45%) |  |
| **PR** |  |  | 0.73 |
| Low | 413 (53%) | 118 (54%) |  |
| High | 373 (47%) | 101 (46%) |  |
| **HER2** |  |  | 0.32 |
| 0 | 506 (64%) | 133 (61%) |  |
| Low | 280 (36%) | 86 (39%) |  |
| **Neo or Adjuvant Chemotherapy** | 379 (48%) | 115 (53%) | 0.26 |
| **Adjuvant Endocrine Therapy** | 537 (68%) | 166 (76%) | ***0.033*** |
| **Bone-only Disease** | 366 (47%) | 118 (54%) | 0.055 |
| **Bone Metastasis Number** |  |  | 0.19 |
| Low | 409 (52%) | 125 (57%) |  |
| High | 377 (48%) | 94 (43%) |  |
| **Visceral Metastasis** | 380 (48%) | 97 (44%) | 0.29 |
| **Setting** |  |  | 0.48 |
| Endocrine Resistant | 398 (51%) | 105 (48%) |  |
| Endocrine Sensitive | 388 (49%) | 114 (52%) |  |
| **Endocrine Therapy** |  |  | 0.85 |
| Aromatase Inhibitor | 526 (67%) | 148 (68%) |  |
| Fulvestrant | 260 (33%) | 71 (32%) |  |

**Supplementary Table 4:** Clinic-pathological features of patients treated with Ribociclib or Palbociclib (PSM cohort).

| **Characteristic** | **Ribociclib N = 357 (50%)** | **Palbociclib N = 357 (50%)** | **p-value** |
| --- | --- | --- | --- |
| **Premenopausal State** | 92 (26%) | 96 (27%) | 0.73 |
| **Age** |  |  | >0.99 |
| <65 | 234 (66%) | 234 (66%) |  |
| >/=65 | 123 (34%) | 123 (34%) |  |
| **Performance Status** |  |  | 0.59 |
| ECOG 0 | 324 (91%) | 328 (92%) |  |
| ECOG 1 | 33 (9.2%) | 29 (8.1%) |  |
| **Histology** |  |  | 0.98 |
| Ductal | 261 (73%) | 263 (74%) |  |
| Lobular | 78 (22%) | 77 (22%) |  |
| Other | 18 (5.0%) | 17 (4.8%) |  |
| **Ki67** |  |  | 0.94 |
| High | 185 (52%) | 184 (52%) |  |
| Low | 172 (48%) | 173 (48%) |  |
| **Grading** |  |  | 0.69 |
| G1/G2 | 240 (67%) | 245 (69%) |  |
| G3 | 117 (33%) | 112 (31%) |  |
| **ER** |  |  | >0.99 |
| Low | 227 (64%) | 227 (64%) |  |
| High | 130 (36%) | 130 (36%) |  |
| **PR** |  |  | 0.94 |
| Low | 172 (48%) | 173 (48%) |  |
| High | 185 (52%) | 184 (52%) |  |
| **HER2** |  |  | 0.58 |
| 0 | 232 (65%) | 239 (67%) |  |
| Low | 125 (35%) | 118 (33%) |  |
| **Neo or Adjuvant Chemotherapy** | 143 (40%) | 150 (42%) | 0.59 |
| **Adjuvant Endocrine Therapy** | 192 (54%) | 197 (55%) | 0.71 |
| **Bone-only Disease** | 184 (52%) | 184 (52%) | >0.99 |
| **Bone Metastasis Number** |  |  | 0.65 |
| Low | 171 (48%) | 177 (50%) |  |
| High | 186 (52%) | 180 (50%) |  |
| **Visceral Metastasis** | 150 (42%) | 155 (43%) | 0.71 |
| **Setting** |  |  | 0.36 |
| Endocrine Resistant | 96 (27%) | 107 (30%) |  |
| Endocrine Sensitive | 261 (73%) | 250 (70%) |  |
| **Endocrine Therapy** |  |  | 0.42 |
| Aromatase Inhibitor | 249 (70%) | 239 (67%) |  |
| Fulvestrant | 108 (30%) | 118 (33%) |  |

**Supplementary Table 5:** Clinic-pathological features of patients treated with Ribociclib or Abemaciclib (PSM cohort).

| **Characteristic** | **Ribociclib N = 167 (50%)** | **Abemaciclib N = 167 (50%)** | **p-value** |
| --- | --- | --- | --- |
| **Premenopausal State** | 37 (22%) | 37 (22%) | >0.99 |
| **Age** |  |  | 0.82 |
| <65 | 99 (59%) | 97 (58%) |  |
| >/=65 | 68 (41%) | 70 (42%) |  |
| **Performance Status** |  |  | 0.41 |
| ECOG 0 | 143 (86%) | 148 (89%) |  |
| ECOG 1 | 24 (14%) | 19 (11%) |  |
| **Histology** |  |  | 0.87 |
| Ductal | 123 (74%) | 119 (71%) |  |
| Lobular | 36 (22%) | 40 (24%) |  |
| Other | 8 (4.8%) | 8 (4.8%) |  |
| **Ki67** |  |  | 0.66 |
| High | 81 (49%) | 77 (46%) |  |
| Low | 86 (51%) | 90 (54%) |  |
| **Grading** |  |  | 0.72 |
| G1/G2 | 114 (68%) | 50 (30%) |  |
| G3 | 53 (32%) | 117 (70%) |  |
| **ER** |  |  | >0.99 |
| Low | 93 (56%) | 93 (56%) |  |
| High | 74 (44%) | 74 (44%) |  |
| **PR** |  |  | >0.99 |
| Low | 86 (51%) | 86 (51%) |  |
| High | 81 (49%) | 81 (49%) |  |
| **HER2** |  |  | 0.82 |
| 0 | 102 (61%) | 100 (60%) |  |
| Low | 65 (39%) | 67 (40%) |  |
| **Neo or Adjuvant Chemotherapy** | 86 (51%) | 86 (51%) | >0.99 |
| **Adjuvant Endocrine Therapy** | 119 (71%) | 118 (71%) | 0.9 |
| **Bone-only Disease** | 85 (51%) | 90 (54%) | 0.58 |
| **Bone Metastasis Number** |  |  | 0.51 |
| Low | 85 (51%) | 91 (54%) |  |
| High | 82 (49%) | 76 (46%) |  |
| **Visceral Metastasis** | 76 (46%) | 73 (44%) | 0.74 |
| **Setting** |  |  | 0.91 |
| Endocrine Resistant | 63 (38%) | 64 (38%) |  |
| Endocrine Sensitive | 104 (62%) | 103 (62%) |  |
| **Endocrine Therapy** |  |  | >0.99 |
| Aromatase Inhibitor | 112 (67%) | 112 (67%) |  |
| Fulvestrant | 55 (33%) | 55 (33%) |  |

**Supplementary Table 6:** Clinic-pathological features of patients treated with Palbociclib or Abemaciclib (PSM)

| **Characteristic** | **Abemaciclib N = 218 (50%)** | **Palbociclib N = 218 (50%)** | **p-value** |
| --- | --- | --- | --- |
| **Premenopausal State** | 45 (21%) | 39 (18%) | 0.47 |
| **Age** |  |  | 0.92 |
| <65 | 124 (57%) | 125 (57%) |  |
| >/=65 | 94 (43%) | 93 (43%) |  |
| **Performance Status** |  |  | 0.79 |
| ECOG 0 | 184 (84%) | 186 (85%) |  |
| ECOG 1 | 34 (16%) | 32 (15%) |  |
| **Histology** |  |  | 0.63 |
| Ductal | 159 (73%) | 166 (76%) |  |
| Lobular | 47 (22%) | 39 (18%) |  |
| Other | 12 (5.5%) | 13 (6.0%) |  |
| **Ki67** |  |  | 0.63 |
| High | 104 (48%) | 109 (50%) |  |
| Low | 114 (52%) | 109 (50%) |  |
| **Grading** |  |  | 0.46 |
| G1/G2 | 150 (69%) | 157 (72%) |  |
| G3 | 68 (31%) | 61 (28%) |  |
| **ER** |  |  | 0.5 |
| Low | 120 (55%) | 113 (52%) |  |
| High | 98 (45%) | 105 (48%) |  |
| **PR** |  |  | 0.33 |
| Low | 118 (54%) | 128 (59%) |  |
| High | 100 (46%) | 90 (41%) |  |
| **HER2** |  |  | 0.7 |
| 0 | 133 (61%) | 129 (59%) |  |
| Low | 85 (39%) | 89 (41%) |  |
| **Neo or Adjuvant Chemotherapy** | 114 (52%) | 115 (53%) | 0.92 |
| **Adjuvant Endocrine Therapy** | 165 (76%) | 169 (78%) | 0.65 |
| **Bone-only Disease** | 117 (54%) | 120 (55%) | 0.77 |
| **Bone Metastasis Number** |  |  | 0.85 |
| Low | 124 (57%) | 122 (56%) |  |
| High | 94 (43%) | 96 (44%) |  |
| **Visceral Metastasis** | 97 (44%) | 93 (43%) | 0.7 |
| **Setting** |  |  | 0.57 |
| Endocrine Resistant | 105 (48%) | 111 (51%) |  |
| Endocrine Sensitive | 113 (52%) | 107 (49%) |  |
| **Endocrine Therapy** |  |  | 0.84 |
| Aromatase Inhibitor | 147 (67%) | 149 (68%) |  |
| Fulvestrant | 71 (33%) | 69 (32%) |  |

**Supplementary Table 7:** Clinic-pathological features of patients treated with Palbociclib or Ribociclib (IPTW cohort).

| **Characteristic** | **Palbociclib N = 1140 (50%)** | **Ribociclib N = 1140 (50%)** | **p-value** |
| --- | --- | --- | --- |
| **Premenopausal State** | 247 (22%) | 260 (23%) | 0.51 |
| **Age** |  |  | 0.47 |
| <65 | 684 (60%) | 701 (61%) |  |
| >/=65 | 456 (40%) | 439 (39%) |  |
| **Performance Status** |  |  | 0.95 |
| ECOG 0 | 990 (87%) | 989 (87%) |  |
| ECOG 1 | 150 (13%) | 151 (13%) |  |
| **Histology** |  |  | 0.98 |
| Ductal | 825 (72%) | 821 (72%) |  |
| Lobular | 244 (21%) | 246 (22%) |  |
| Other | 71 (6.2%) | 73 (6.4%) |  |
| **Ki67** |  |  | 0.97 |
| High | 583 (51%) | 582 (51%) |  |
| Low | 557 (49%) | 558 (49%) |  |
| **Grading** |  |  | 0.26 |
| G1/G2 | 772 (68%) | 797 (70%) |  |
| G3 | 368 (32%) | 343 (30%) |  |
| **ER** |  |  | 0.76 |
| Low | 685 (60%) | 692 (61%) |  |
| High | 455 (40%) | 448 (39%) |  |
| **PR** |  |  | 0.29 |
| Low | 582 (51%) | 607 (53%) |  |
| High | 558 (49%) | 533 (47%) |  |
| **HER2** |  |  | 0.6 |
| 0 | 745 (65%) | 757 (66%) |  |
| Low | 395 (35%) | 383 (34%) |  |
| **Neo or Adjuvant Chemotherapy** | 499 (44%) | 510 (45%) | 0.64 |
| **Adjuvant Endocrine Therapy** | 699 (61%) | 689 (60%) | 0.67 |
| **Bone-only Disease** | 565 (50%) | 556 (49%) | 0.71 |
| **Bone Metastasis Number** |  |  | 0.8 |
| Low | 579 (51%) | 585 (51%) |  |
| High | 561 (49%) | 555 (49%) |  |
| **Visceral Metastasis** | 507 (44%) | 513 (45%) | 0.8 |
| **Setting** |  |  | 0.32 |
| Endocrine Resistant | 449 (39%) | 426 (37%) |  |
| Endocrine Sensitive | 691 (61%) | 714 (63%) |  |
| **Endocrine Therapy** |  |  | 0.4 |
| Aromatase Inhibitor | 771 (68%) | 752 (66%) |  |
| Fulvestrant | 369 (32%) | 388 (34%) |  |

**Supplementary Table 8:** Clinic-pathological features of patients treated with Ribociclib or Abemaciclib (IPTW cohort).

| **Characteristic** | **Ribociclib N = 335 (50%)** | **Abemaciclib N = 335 (50%)** | **p-value** |
| --- | --- | --- | --- |
| **Premenopausal State** | 75 (22%) | 73 (22%) | 0.85 |
| **Age** |  |  | 0.69 |
| <65 | 199 (59%) | 194 (58%) |  |
| >/=65 | 136 (41%) | 141 (42%) |  |
| **Performance Status** |  |  | 0.3 |
| ECOG 0 | 287 (86%) | 296 (88%) |  |
| ECOG 1 | 48 (14%) | 39 (12%) |  |
| **Histology** |  |  | 0.76 |
| Ductal | 246 (73%) | 238 (71%) |  |
| Lobular | 73 (22%) | 81 (24%) |  |
| Other | 16 (4.8%) | 16 (4.8%) |  |
| **Ki67** |  |  | 0.54 |
| High | 162 (48%) | 154 (46%) |  |
| Low | 173 (52%) | 181 (54%) |  |
| **Grading** |  |  | 0.56 |
| G1/G2 | 229 (68%) | 236 (70%) |  |
| G3 | 106 (32%) | 99 (30%) |  |
| **ER** |  |  | 0.94 |
| Low | 187 (56%) | 186 (56%) |  |
| High | 148 (44%) | 149 (44%) |  |
| **PR** |  |  | >0.99 |
| Low | 172 (51%) | 172 (51%) |  |
| High | 163 (49%) | 163 (49%) |  |
| **HER2** |  |  | 0.75 |
| 0 | 204 (61%) | 200 (60%) |  |
| Low | 131 (39%) | 135 (40%) |  |
| **Neo or Adjuvant Chemotherapy** | 172 (51%) | 171 (51%) | 0.94 |
| **Adjuvant Endocrine Therapy** | 238 (71%) | 236 (70%) | 0.87 |
| **Bone-only Disease** | 170 (51%) | 179 (53%) | 0.49 |
| **Bone Metastasis Number** |  |  | 0.39 |
| Low | 171 (51%) | 182 (54%) |  |
| High | 164 (49%) | 153 (46%) |  |
| **Visceral Metastasis** | 153 (46%) | 146 (44%) | 0.59 |
| **Setting** |  |  | >0.99 |
| Endocrine Resistant | 127 (38%) | 127 (38%) |  |
| Endocrine Sensitive | 208 (62%) | 208 (62%) |  |
| **Endocrine Therapy** |  |  | 0.93 |
| Aromatase Inhibitor | 225 (67%) | 226 (67%) |  |
| Fulvestrant | 110 (33%) | 109 (33%) |  |

**Supplementary Table 9:** Clinic-pathological features of patients treated with Abemaciclib or Palbociclib (IPTW)

| **Characteristic** | **Abemaciclib N = 881 (50%)** | **Palbociclib N = 881 (50%)** | **p-value** |
| --- | --- | --- | --- |
| **Premenopausal State** | 176 (20%) | 177 (20%) | 0.95 |
| **Age** |  |  | 0.63 |
| <65 | 494 (56%) | 504 (57%) |  |
| >/=65 | 387 (44%) | 377 (43%) |  |
| **Performance Status** |  |  | 0.57 |
| ECOG 0 | 729 (83%) | 738 (84%) |  |
| ECOG 1 | 152 (17%) | 143 (16%) |  |
| **Histology** |  |  | 0.88 |
| Ductal | 640 (73%) | 638 (72%) |  |
| Lobular | 187 (21%) | 193 (22%) |  |
| Other | 54 (6.1%) | 50 (5.7%) |  |
| **Ki67** |  |  | 0.85 |
| High | 422 (48%) | 426 (48%) |  |
| Low | 459 (52%) | 455 (52%) |  |
| **Grading** |  |  | 0.8 |
| G1/G2 | 610 (69%) | 605 (69%) |  |
| G3 | 271 (31%) | 276 (31%) |  |
| **ER** |  |  | 0.77 |
| Low | 490 (56%) | 484 (55%) |  |
| High | 391 (44%) | 397 (45%) |  |
| **PR** |  |  | 0.74 |
| Low | 478 (54%) | 471 (53%) |  |
| High | 403 (46%) | 410 (47%) |  |
| **HER2** |  |  | 0.92 |
| 0 | 534 (61%) | 532 (60%) |  |
| Low | 347 (39%) | 349 (40%) |  |
| **Neo or Adjuvant Chemotherapy** | 453 (51%) | 443 (50%) | 0.63 |
| **Adjuvant Endocrine Therapy** | 669 (76%) | 647 (73%) | 0.23 |
| **Bone-only Disease** | 446 (51%) | 454 (52%) | 0.7 |
| **Bone Metastasis Number** |  |  | 0.67 |
| Low | 479 (54%) | 488 (55%) |  |
| High | 402 (46%) | 393 (45%) |  |
| **Visceral Metastasis** | 388 (44%) | 387 (44%) | 0.96 |
| **Setting** |  |  | 0.45 |
| Endocrine Resistant | 423 (48%) | 407 (46%) |  |
| Endocrine Sensitive | 458 (52%) | 474 (54%) |  |
| **Endocrine Therapy** |  |  | 0.65 |
| Aromatase Inhibitor | 599 (68%) | 590 (67%) |  |
| Fulvestrant | 282 (32%) | 291 (33%) |  |

**Supplementary Table 10:** Sensitivity analysis for rwPFS

| **rwPFS** | **all** | **HR (univariable)** | **HR (multivariable)** |
| --- | --- | --- | --- |
| **Complete Population** |  |  |  |
| Palbociclib | 786 (56.2) | - | - |
| Abemaciclib | 219 (15.7) | 0.70 (0.56-0.86, p=0.001) | 0.72 (0.58-0.90, p=0.004) |
| Ribociclib | 394 (28.2) | 0.67 (0.57-0.79, p<0.001) | 0.77 (0.65-0.92, p=0.004) |
| **Excluding Center 1** |  |  |  |
| Palbociclib | 726 (55.2) | - | - |
| Abemaciclib | 216 (16.4) | 0.65 (0.52-0.81, p<0.001) | 0.66 (0.53-0.83, p<0.001) |
| Ribociclib | 374 (28.4) | 0.65 (0.55-0.76, p<0.001) | 0.73 (0.61-0.87, p<0.001) |
| **Excluding Center 2** |  |  |  |
| Palbociclib | 759 (55.5) | - | - |
| Abemaciclib | 219 (15.9) | 0.70 (0.56-0.87, p=0.001) | 0.73 (0.59-0.91, p=0.005) |
| Ribociclib | 394 (28.6) | 0.67 (0.57-0.79, p<0.001) | 0.77 (0.65-0.92, p=0.004) |
| **Excluding Center 3** |  |  |  |
| Palbociclib | 759 (57.0) | - | - |
| Abemaciclib | 212 (15.9) | 0.68 (0.55-0.85, p=0.001) | 0.72 (0.58-0.90, p=0.003) |
| Ribociclib | 361 (27.1) | 0.68 (0.57-0.80, p<0.001) | 0.78 (0.65-0.93, p=0.006) |
| **Excluding Center 4** |  |  |  |
| Palbociclib | 761 (56.2) | - | - |
| Abemaciclib | 211 (15.6) | 0.70 (0.56-0.88, p=0.002) | 0.73 (0.58-0.91, p=0.005) |
| Ribociclib | 382 (28.2) | 0.68 (0.58-0.80, p<0.001) | 0.80 (0.67-0.95, p=0.011) |
| **Excluding Center 5** |  |  |  |
| Palbociclib | 758 (55.3) | - | - |
| Abemaciclib | 219 (16.0) | 0.71 (0.57-0.88, p=0.002) | 0.74 (0.59-0.92, p=0.006) |
| Ribociclib | 393 (28.7) | 0.69 (0.58-0.81, p<0.001) | 0.79 (0.67-0.94, p=0.008) |
| **Excluding Center 6** |  |  |  |
| Palbociclib | 782 (56.1) | - | - |
| Abemaciclib | 219 (15.7) | 0.70 (0.56-0.87, p=0.001) | 0.73 (0.58-0.91, p=0.004) |
| Ribociclib | 394 (28.2) | 0.68 (0.58-0.80, p<0.001) | 0.78 (0.66-0.93, p=0.005) |
| **Excluding Center 7** |  |  |  |
| Palbociclib | 770 (56.0) | - | - |
| Abemaciclib | 217 (15.8) | 0.69 (0.56-0.86, p=0.001) | 0.72 (0.58-0.91, p=0.004) |
| Ribociclib | 387 (28.2) | 0.66 (0.56-0.78, p<0.001) | 0.77 (0.65-0.92, p=0.003) |
| **Excluding Center 8** |  |  |  |
| Palbociclib | 751 (55.2) | - | - |
| Abemaciclib | 218 (16.0) | 0.69 (0.55-0.86, p=0.001) | 0.71 (0.57-0.89, p=0.003) |
| Ribociclib | 391 (28.8) | 0.67 (0.57-0.79, p<0.001) | 0.77 (0.65-0.92, p=0.004) |
| **Excluding Center 9** |  |  |  |
| Palbociclib | 707 (55.4) | - | - |
| Abemaciclib | 215 (15.7) | 0.73 (0.59-0.92, p=0.007) | 0.77 (0.62-0.97, p=0.026) |
| Ribociclib | 369 (28.9) | 0.69 (0.59-0.82, p<0.001) | 0.81 (0.67-0.96, p=0.019) |
| **Excluding Center 10** |  |  |  |
| Palbociclib | 765 (56.8) | - | - |
| Abemaciclib | 208 (15.4) | 0.66 (0.52-0.83, p<0.001) | 0.68 (0.54-0.86, p=0.001) |
| Ribociclib | 375 (27.8) | 0.68 (0.57-0.80, p<0.001) | 0.78 (0.65-0.93, p=0.005) |
| **Excluding Center 11** |  |  |  |
| Palbociclib | 771 (56.9) | - | - |
| Abemaciclib | 204 (15.1) | 0.74 (0.60-0.93, p=0.009) | 0.77 (0.61-0.96, p=0.019) |
| Ribociclib | 379 (28.0) | 0.69 (0.59-0.82, p<0.001) | 0.80 (0.67-0.95, p=0.010) |
| **Excluding Center 12** |  |  |  |
| Palbociclib | 761 (56.5) | - | - |
| Abemaciclib | 204 (15.2) | 0.69 (0.55-0.86, p=0.001) | 0.72 (0.57-0.90, p=0.005) |
| Ribociclib | 381 (28.3) | 0.67 (0.57-0.79, p<0.001) | 0.78 (0.65-0.93, p=0.006) |
| **Excluding Center 13** |  |  |  |
| Palbociclib | 784 (56.9) | - | - |
| Abemaciclib | 204 (14.8) | 0.71 (0.57-0.88, p=0.002) | 0.74 (0.59-0.92, p=0.007) |
| Ribociclib | 391 (28.4) | 0.67 (0.57-0.79, p<0.001) | 0.76 (0.64-0.91, p=0.002) |
| **Excluding Center 14** | | | |
| Palbociclib | 774 (57.5) | - | - |
| Abemaciclib | 205 (15.2) | 0.69 (0.55-0.86, p=0.001) | 0.71 (0.57-0.89, p=0.003) |
| Ribociclib | 366 (27.2) | 0.68 (0.57-0.80, p<0.001) | 0.78 (0.65-0.93, p=0.005) |
| **Excluding Center 15** |  |  |  |
| Palbociclib | 737 (55.5) | - | - |
| Abemaciclib | 207 (15.6) | 0.70 (0.56-0.87, p=0.001) | 0.74 (0.59-0.92, p=0.008) |
| Ribociclib | 385 (29.0) | 0.67 (0.57-0.79, p<0.001) | 0.77 (0.65-0.92, p=0.004) |
| **Excluding Center 16** |  |  |  |
| Palbociclib | 756 (56.5) | - | - |
| Abemaciclib | 201 (15.0) | 0.73 (0.58-0.91, p=0.005) | 0.78 (0.62-0.97, p=0.028) |
| Ribociclib | 381 (28.5) | 0.67 (0.57-0.79, p<0.001) | 0.78 (0.65-0.93, p=0.005) |
| **Excluding Center 17** | | | |
| Palbociclib | 774 (56.9) | - | - |
| Abemaciclib | 211 (15.5) | 0.68 (0.54-0.84, p=0.001) | 0.71 (0.57-0.88, p=0.002) |
| Ribociclib | 375 (27.6) | 0.66 (0.56-0.77, p<0.001) | 0.76 (0.64-0.91, p=0.002) |
| **Excluding Center 18** | | | |
| Palbociclib | 697 (55.7) | - | - |
| Abemaciclib | 196 (15.7) | 0.77 (0.61-0.96, p=0.019) | 0.79 (0.63-0.99, p=0.043) |
| Ribociclib | 358 (28.6) | 0.65 (0.55-0.77, p<0.001) | 0.74 (0.62-0.89, p=0.001) |
| **Excluding Center 19** | | | |
| Palbociclib | 760 (55.7) | - | - |
| Abemaciclib | 215 (15.8) | 0.71 (0.57-0.88, p=0.002) | 0.73 (0.59-0.91, p=0.006) |
| Ribociclib | 389 (28.5) | 0.68 (0.58-0.81, p<0.001) | 0.78 (0.66-0.94, p=0.007) |
| **Excluding Center 20** | | | |
| Palbociclib | 710 (55.0) | - | - |
| Abemaciclib | 214 (16.6) | 0.72 (0.58-0.90, p=0.004) | 0.75 (0.60-0.94, p=0.013) |
| Ribociclib | 368 (28.5) | 0.72 (0.61-0.85, p<0.001) | 0.83 (0.70-1.00, p=0.044) |
| **Excluding Center 21** | | | |
| Palbociclib | 778 (56.7) | - | - |
| Abemaciclib | 214 (15.6) | 0.68 (0.55-0.85, p=0.001) | 0.71 (0.57-0.89, p=0.002) |
| Ribociclib | 379 (27.6) | 0.67 (0.57-0.79, p<0.001) | 0.76 (0.64-0.91, p=0.002) |
| **Excluding Center 22** | | | |
| Palbociclib | 767 (56.0) | - | - |
| Abemaciclib | 219 (16.0) | 0.68 (0.55-0.85, p=0.001) | 0.72 (0.58-0.89, p=0.003) |
| Ribociclib | 383 (28.0) | 0.66 (0.56-0.78, p<0.001) | 0.77 (0.64-0.92, p=0.003) |
| **Excluding Center 23** | | | |
| Palbociclib | 778 (56.9) | - | - |
| Abemaciclib | 215 (15.7) | 0.70 (0.57-0.87, p=0.002) | 0.73 (0.59-0.91, p=0.005) |
| Ribociclib | 374 (27.4) | 0.67 (0.57-0.79, p<0.001) | 0.77 (0.64-0.92, p=0.003) |
| **Excluding Center 24** |  |  |  |
| Palbociclib | 686 (56.8) | - | - |
| Abemaciclib | 189 (15.6) | 0.60 (0.47-0.77, p<0.001) | 0.60 (0.47-0.77, p<0.001) |
| Ribociclib | 333 (27.6) | 0.67 (0.56-0.80, p<0.001) | 0.75 (0.62-0.90, p=0.003) |

**Supplementary Table 11:** Sensitivity analysis for OS

| **OS** | **all** | **HR (univariable)** | **HR (multivariable)** |
| --- | --- | --- | --- |
| **Complete Population** |  |  |  |
| Palbociclib | 786 (56.2) | - | - |
| Abemaciclib | 219 (15.7) | 0.62 (0.46-0.85, p=0.003) | 0.68 (0.49-0.92, p=0.014) |
| Ribociclib | 394 (28.2) | 0.62 (0.50-0.78, p<0.001) | 0.71 (0.56-0.89, p=0.003) |
| **Excluding Center 1** |  |  |  |
| Palbociclib | 726 (55.2) | - | - |
| Abemaciclib | 216 (16.4) | 0.60 (0.43-0.82, p=0.001) | 0.63 (0.46-0.87, p=0.005) |
| Ribociclib | 374 (28.4) | 0.60 (0.48-0.76, p<0.001) | 0.68 (0.53-0.86, p=0.002) |
| **Excluding Center 2** |  |  |  |
| Palbociclib | 759 (55.5) | - | - |
| Abemaciclib | 219 (15.9) | 0.63 (0.46-0.86, p=0.003) | 0.68 (0.50-0.93, p=0.017) |
| Ribociclib | 394 (28.6) | 0.63 (0.50-0.78, p<0.001) | 0.71 (0.56-0.90, p=0.004) |
| **Excluding Center 3** |  |  |  |
| Palbociclib | 759 (57.0) | - | - |
| Abemaciclib | 212 (15.9) | 0.61 (0.45-0.84, p=0.002) | 0.67 (0.49-0.92, p=0.012) |
| Ribociclib | 361 (27.1) | 0.63 (0.51-0.79, p<0.001) | 0.71 (0.56-0.89, p=0.004) |
| **Excluding Center 4** |  |  |  |
| Palbociclib | 761 (56.2) | - | - |
| Abemaciclib | 211 (15.6) | 0.65 (0.48-0.89, p=0.007) | 0.71 (0.52-0.97, p=0.031) |
| Ribociclib | 382 (28.2) | 0.62 (0.49-0.77, p<0.001) | 0.72 (0.57-0.91, p=0.006) |
| **Excluding Center 5** |  |  |  |
| Palbociclib | 758 (55.3) | - | - |
| Abemaciclib | 219 (16.0) | 0.64 (0.47-0.88, p=0.005) | 0.70 (0.51-0.96, p=0.027) |
| Ribociclib | 393 (28.7) | 0.64 (0.51-0.80, p<0.001) | 0.73 (0.58-0.92, p=0.007) |
| **Excluding Center 6** |  |  |  |
| Palbociclib | 782 (56.1) | - | - |
| Abemaciclib | 219 (15.7) | 0.63 (0.46-0.85, p=0.003) | 0.68 (0.50-0.93, p=0.014) |
| Ribociclib | 394 (28.2) | 0.62 (0.50-0.78, p<0.001) | 0.71 (0.56-0.89, p=0.003) |
| **Excluding Center 7** |  |  |  |
| Palbociclib | 770 (56.0) | - | - |
| Abemaciclib | 217 (15.8) | 0.62 (0.45-0.84, p=0.002) | 0.67 (0.49-0.92, p=0.012) |
| Ribociclib | 387 (28.2) | 0.61 (0.49-0.76, p<0.001) | 0.70 (0.56-0.88, p=0.003) |
| **Excluding Center 8** |  |  |  |
| Palbociclib | 751 (55.2) | - | - |
| Abemaciclib | 218 (16.0) | 0.61 (0.45-0.84, p=0.002) | 0.66 (0.49-0.91, p=0.010) |
| Ribociclib | 391 (28.8) | 0.62 (0.49-0.77, p<0.001) | 0.70 (0.56-0.88, p=0.003) |
| **Excluding Center 9** |  |  |  |
| Palbociclib | 707 (55.4) | - | - |
| Abemaciclib | 215 (15.7) | 0.68 (0.49-0.93, p=0.015) | 0.72 (0.53-0.99, p=0.044) |
| Ribociclib | 369 (28.9) | 0.66 (0.53-0.82, p<0.001) | 0.75 (0.59-0.95, p=0.017) |
| **Excluding Center 10** |  |  |  |
| Palbociclib | 765 (56.8) | - | - |
| Abemaciclib | 208 (15.4) | 0.58 (0.42-0.80, p=0.001) | 0.63 (0.45-0.87, p=0.006) |
| Ribociclib | 375 (27.8) | 0.62 (0.49-0.77, p<0.001) | 0.70 (0.55-0.88, p=0.003) |
| **Excluding Center 11** |  |  |  |
| Palbociclib | 771 (56.9) | - | - |
| Abemaciclib | 204 (15.1) | 0.66 (0.48-0.90, p=0.009) | 0.70 (0.51-0.96, p=0.027) |
| Ribociclib | 379 (28.0) | 0.62 (0.50-0.78, p<0.001) | 0.70 (0.55-0.88, p=0.003) |
| **Excluding Center 12** |  |  |  |
| Palbociclib | 761 (56.5) | - | - |
| Abemaciclib | 204 (15.2) | 0.62 (0.45-0.86, p=0.004) | 0.67 (0.49-0.93, p=0.016) |
| Ribociclib | 381 (28.3) | 0.61 (0.49-0.76, p<0.001) | 0.70 (0.55-0.88, p=0.002) |
| **Excluding Center 13** |  |  |  |
| Palbociclib | 784 (56.9) | - | - |
| Abemaciclib | 204 (14.8) | 0.62 (0.45-0.85, p=0.003) | 0.68 (0.49-0.93, p=0.018) |
| Ribociclib | 391 (28.4) | 0.61 (0.49-0.76, p<0.001) | 0.68 (0.54-0.86, p=0.001) |
| **Excluding Center 14** | | | |
| Palbociclib | 774 (57.5) | - | - |
| Abemaciclib | 205 (15.2) | 0.63 (0.46-0.86, p=0.004) | 0.69 (0.50-0.94, p=0.021) |
| Ribociclib | 366 (27.2) | 0.61 (0.49-0.77, p<0.001) | 0.69 (0.55-0.88, p=0.003) |
| **Excluding Center 15** |  |  |  |
| Palbociclib | 737 (55.5) | - | - |
| Abemaciclib | 207 (15.6) | 0.62 (0.46-0.85, p=0.003) | 0.68 (0.49-0.93, p=0.015) |
| Ribociclib | 385 (29.0) | 0.63 (0.50-0.78, p<0.001) | 0.70 (0.55-0.88, p=0.003) |
| **Excluding Center 16** |  |  |  |
| Palbociclib | 756 (56.5) | - | - |
| Abemaciclib | 201 (15.0) | 0.67 (0.49-0.91, p=0.011) | 0.73 (0.54-1.00, p=0.048) |
| Ribociclib | 381 (28.5) | 0.62 (0.50-0.78, p<0.001) | 0.73 (0.58-0.91, p=0.006) |
| **Excluding Center 17** | | | |
| Palbociclib | 774 (56.9) | - | - |
| Abemaciclib | 211 (15.5) | 0.59 (0.43-0.82, p=0.001) | 0.65 (0.47-0.89, p=0.008) |
| Ribociclib | 375 (27.6) | 0.61 (0.49-0.76, p<0.001) | 0.69 (0.54-0.87, p=0.002) |
| **Excluding Center 18** | | | |
| Palbociclib | 697 (55.7) | - | - |
| Abemaciclib | 196 (15.7) | 0.68 (0.50-0.94, p=0.020) | 0.72 (0.52-1.00, p=0.049) |
| Ribociclib | 358 (28.6) | 0.61 (0.48-0.77, p<0.001) | 0.65 (0.51-0.83, p=0.001) |
| **Excluding Center 19** | | | |
| Palbociclib | 760 (55.7) | - | - |
| Abemaciclib | 215 (15.8) | 0.62 (0.45-0.85, p=0.003) | 0.67 (0.49-0.92, p=0.013) |
| Ribociclib | 389 (28.5) | 0.63 (0.50-0.79, p<0.001) | 0.71 (0.56-0.90, p=0.004) |
| **Excluding Center 20** | | | |
| Palbociclib | 710 (55.0) | - | - |
| Abemaciclib | 214 (16.6) | 0.65 (0.47-0.89, p=0.007) | 0.70 (0.51-0.96, p=0.027) |
| Ribociclib | 368 (28.5) | 0.68 (0.55-0.85, p=0.001) | 0.77 (0.61-0.98, p=0.032) |
| **Excluding Center 21** | | | |
| Palbociclib | 778 (56.7) | - | - |
| Abemaciclib | 214 (15.6) | 0.62 (0.45-0.85, p=0.003) | 0.67 (0.49-0.91, p=0.011) |
| Ribociclib | 379 (27.6) | 0.64 (0.51-0.80, p<0.001) | 0.72 (0.57-0.90, p=0.005) |
| **Excluding Center 22** | | | |
| Palbociclib | 767 (56.0) | - | - |
| Abemaciclib | 219 (16.0) | 0.61 (0.45-0.83, p=0.002) | 0.66 (0.49-0.91, p=0.010) |
| Ribociclib | 383 (28.0) | 0.62 (0.50-0.77, p<0.001) | 0.70 (0.56-0.89, p=0.003) |
| **Excluding Center 23** | | | |
| Palbociclib | 778 (56.9) | - | - |
| Abemaciclib | 215 (15.7) | 0.64 (0.47-0.87, p=0.004) | 0.69 (0.50-0.94, p=0.019) |
| Ribociclib | 374 (27.4) | 0.60 (0.48-0.76, p<0.001) | 0.69 (0.55-0.88, p=0.002) |
| **Excluding Center 24** |  |  |  |
| Palbociclib | 686 (56.8) | - | - |
| Abemaciclib | 189 (15.6) | 0.50 (0.35-0.73, p<0.001) | 0.51 (0.35-0.74, p<0.001) |
| Ribociclib | 333 (27.6) | 0.64 (0.50-0.81, p<0.001) | 0.69 (0.54-0.89, p=0.005) |

**Supplementary Table 12:** Sensitivity analysis stratified in treatment line in calendar time

| **Endpoint** | **Analysis** | **Palb vs Abema HR (95% CI)** | **p (Palb vs Abema)** | **Ribo vs Abema HR (95% CI)** | **p (Ribo vs Abema)** | **Palb vs Ribo HR (95% CI)** | **p (Palb vs Ribo)** | **N** | **Events** |
| --- | --- | --- | --- | --- | --- | --- | --- | --- | --- |
| **rwPFS** | First-line only (+year) | 1.53 (1.17–2.00) | 0.002 | 1.17 (0.88–1.54) | 0.273 | 1.31 (1.08–1.60) | 0.007 | 1142 | 648 |
| **rwPFS** | Second-line only (+year) | 1.56 (1.01–2.41) | 0.046 | 1.40 (0.74–2.65) | 0.296 | 1.11 (0.63–1.97) | 0.719 | 257 | 189 |
| **OS** | First-line only (+year) | 1.72 (1.19–2.49) | 0.004 | 1.06 (0.72–1.57) | 0.773 | 1.63 (1.25–2.11) | <0.001 | 1142 | 399 |
| **OS** | Second-line only (+year) | 1.92 (0.89–4.14) | 0.098 | 1.86 (0.64–5.40) | 0.251 | 1.03 (0.45–2.33) | 0.946 | 257 | 111 |
| **rwPFS** | Initiated ≥2020 (+line,+year) | 1.40 (1.04–1.88) | 0.025 | 1.25 (0.92–1.71) | 0.160 | 1.12 (0.84–1.48) | 0.438 | 666 | 335 |
| **OS** | Initiated ≥2020 (+line,+year) | 1.69 (1.11–2.58) | 0.014 | 1.22 (0.76–1.94) | 0.406 | 1.39 (0.94–2.06) | 0.100 | 666 | 173 |
