# Supplementary material for: Comparative real-world progression free survival of CDK4/6 inhibitors in HR+/HER2− breast cancer patients with bone metastases
Source: Oncologist. 2026 Apr 16;31(5):oyag146. doi: 10.1093/oncolo/oyag146 (PMC13127761; doi:10.1093/oncolo/oyag146)

		Number of Patients		Median rwPFS (months)		Palbociclib vs Abemaciclib		
		Palbociclib	Abemaciclib	Palbociclib	Abemaciclib	HR	(95% CI)	
**Premenopausal State**	Yes	137	46	22	40	2.06	(1.21-3.51)	p = 0.008
	No	649	173	22	29	1.32	(1.04-1.68)	p = 0.021
**Age**	< 65	423	125	22	33	1.40	(1.06-1.86)	p = 0.020
	>/=65	363	94	23	29	1.48	(1.06-2.07)	p = 0.022
**PS**	ECOG 0	650	185	24	35	1.44	(1.13-1.83)	p = 0.003
	ECOG 1	136	34	18	26	1.41	(0.86-2.31)	p = 0.169
**Histology**	Ductal	557	160	22	35	1.72	(1.32-2.25)	p = 0.001
	Lobular	175	47	22	26	1.05	(0.69-1.60)	p = 0.830
**Ki67**	Low	396	114	24	30	1.36	(1.00-1.84)	p = 0.047
	High	390	105	21	36	1.52	(1.11-2.07)	p = 0.009
**Grading**	G1-G2	533	150	24	30	1.42	(1.09-1.86)	p = 0.009
	G3	253	69	20	33	1.47	(1.01-1.13)	p = 0.044
**ER**	Low	443	120	25	28	1.06	(0.81-1.40)	p = 0.665
	High	343	99	19	NR	2.13	(1.50-3.02)	p = 0.001
**PR**	Low	413	118	21	29	1.38	(1.04-1.83)	p = 0.027
	High	373	101	24	35	1.53	(1.10-2.14)	p = 0.012
**HER2**	0	506	133	23	30	1.40	(1.06-1.84)	p = 0.016
	Low	280	86	22	35	1.50	(1.05-2.13)	p = 0.026
**Neo or Adjuvant Chemotherapy**	Yes	379	115	22	28	1.33	(1.00-1.78)	p = 0.047
	No	407	104	23	36	1.59	(1.14-2.21)	p = 0.006
**Adjuvant Endocrine Therapy**	Yes	537	166	22	28	1.28	(1.01-1.62)	p = 0.042
	No	249	53	23	NR	2.47	(1.43-4.27)	p = 0.001
**Bone-only disease**	Yes	366	118	28	35	1.22	(0.89-1.67)	p = 0.209
	No	420	101	17	28	1.59	(1.17-2.15)	p = 0.003
**Bone Metastasis Number**	Low	409	125	24	30	1.36	(1.02-1.82)	p = 0.038
	High	377	94	22	32	1.54	(1.11-2.14)	p = 0.009
**Visceral Metastasis**	Yes	380	97	17	28	1.58	(1.16-2.15)	p = 0.004
	No	406	122	27	35	1.29	(0.95-1.75)	p = 0.100
**Setting**	Endocrine Resistant	398	105	18	27	1.41	(1.05-1.89)	p = 0.023
	Endocrine Sensitive	388	114	27	36	1.45	(1.05-2.00)	p = 0.023
**Endocrine Therapy**	Aromatase Inhibitor	526	148	26	32	1.33	(1.01-1.74)	p = 0.039
	Fulvestrant	260	71	17	29	1.64	(1.14-2.37)	p = 0.007

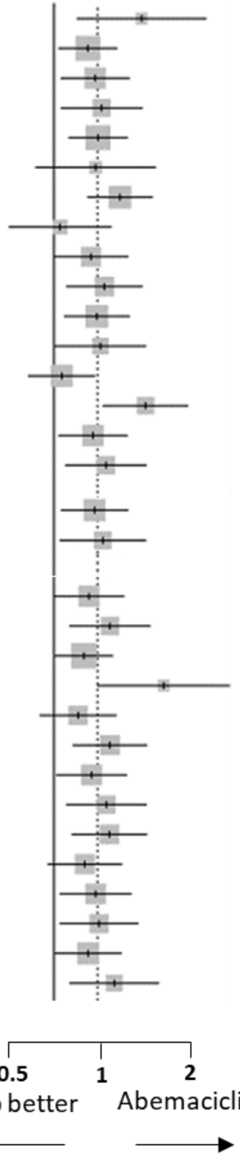

Supplement: oyag146_Supplementary_Data [file oyag146_supplementary_data.zip › Supplementary Figure 1.pdf]
