# Supplementary material for: Comparative real-world progression free survival of CDK4/6 inhibitors in HR+/HER2− breast cancer patients with bone metastases
Source: Oncologist. 2026 Apr 16;31(5):oyag146. doi: 10.1093/oncolo/oyag146 (PMC13127761; doi:10.1093/oncolo/oyag146)

		Number of Patients		Median rwPFS (months)		Palbociclib vs Ribociclib		
		Palbociclib	Ribociclib	Palbociclib	Ribociclib	HR	(95% CI)	
**Premenopausal State**	Yes	137	116	22	3	0.70	(0.66-1.02)	p = 0.035
	No	649	278	22	3	1.52	(1.25-1.82)	p < 0.001
**Age**	< 65	423	267	22	3	1.39	(1.14-1.69)	p = 0.001
	>/=65	363	127	23	3	1.69	(1.27-2.22)	p < 0.001
**PS**	ECOG 0	650	360	24	3	1.49	(1.25-1.75)	p < 0.001
	ECOG 1	136	34	18	1	1.04	(0.66-1.67)	p = 0.851
**Histology**	Ductal	557	294	22	3	1.52	(1.27-1.85)	p < 0.001
	Lobular	175	82	22	3	1.45	(1.00-2.04)	p = 0.034
**Ki67**	Low	396	190	24	3	1.37	(1.09-1.72)	p = 0.009
	High	390	204	21	3	1.59	(1.28-2.00)	p < 0.001
**Grading**	G1-G2	533	268	24	3	1.45	(1.19-1.75)	p < 0.001
	G3	253	126	20	3	1.56	(1.19-2.04)	p = 0.002
**ER**	Low	443	252	25	3	1.35	(1.10-1.67)	p = 0.005
	High	343	142	19	3	1.67	(1.28-2.17)	p < 0.001
**PR**	Low	413	191	21	3	1.35	(1.09-1.69)	p = 0.006
	High	373	203	24	3	1.61	(1.27-2.04)	p < 0.001
**HER2**	0	506	258	23	36	1.47	(1.20-1.79)	p < 0.001
	Low	280	136	22	33	1.47	(1.12-1.96)	p = 0.005
**Neo or Adjuvant Chemotherapy**						1.37	(1.09-1.75)	p = 0.009
	Yes	379	147	22	33	1.54	(1.10-1.92)	p < 0.001
	No	407	247	23	36	1.39	(1.12-1.69)	p = 0.003
**Adjuvant Endocrine Therapy**	Yes	537	203	22	33	1.56	(1.20-5.26)	p = 0.001
	No	249	191	23	36	1.41	(1.10-1.79)	p = 0.006
**Bone-only disease**	Yes	366	208	28	40	1.52	(1.22-1.89)	p < 0.001
	No	420	186	17	27	1.54	(1.22-1.96)	p < 0.001
**Bone Metastasis Number**	Low	409	192	24	37	1.47	(1.18-1.82)	p = 0.001
	High	377	202	22	22	1.49	(1.19-1.89)	p = 0.001
**Visceral Metastasis**	Yes	380	161	17	30	1.43	(1.14-1.79)	p = 0.002
	No	406	233	27	37	1.39	(1.04-1.85)	p = 0.026
**Setting**	Endocrine Resistant	398	96	18	27	1.33	(1.09-1.64)	p = 0.006
	Endocrine Sensitive	388	298	27	36	1.39	(1.14-1.69)	p = 0.001
**Endocrine Therapy**	Aromatase Inhibitor	526	268	26	36	1.67	(1.27-2.17)	p < 0.001
	Fulvestrant	260	126	17	31			

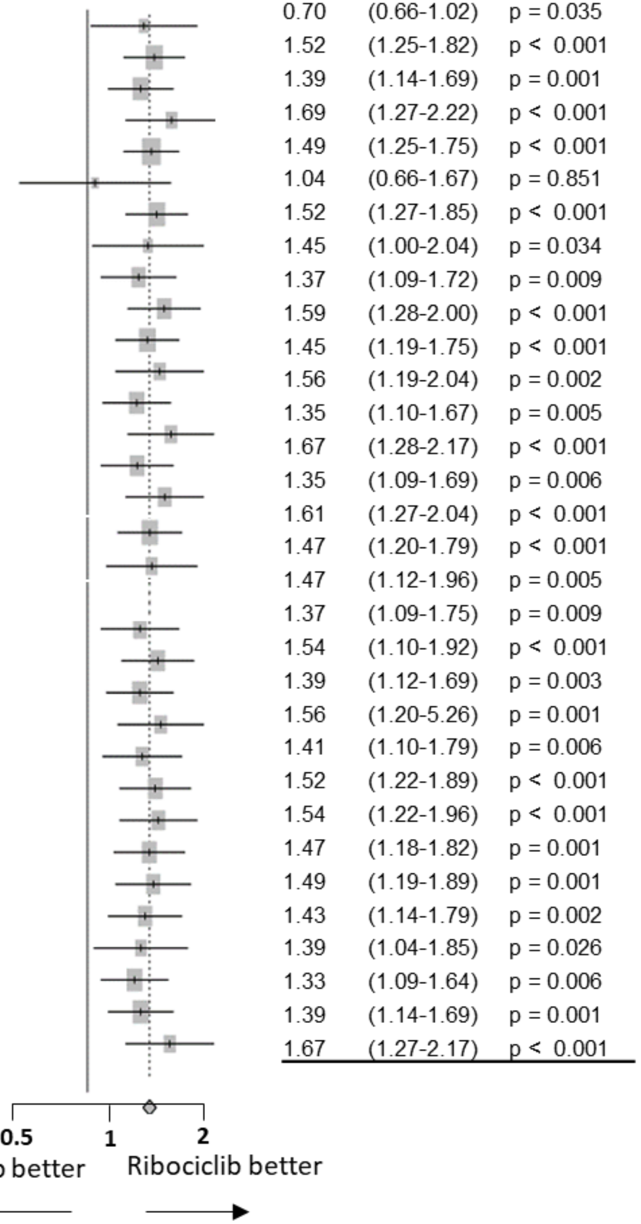

Supplement: oyag146_Supplementary_Data [file oyag146_supplementary_data.zip › Supplementary Figure 2.pdf]
