# Supplementary material for: Comparative real-world progression free survival of CDK4/6 inhibitors in HR+/HER2− breast cancer patients with bone metastases
Source: Oncologist. 2026 Apr 16;31(5):oyag146. doi: 10.1093/oncolo/oyag146 (PMC13127761; doi:10.1093/oncolo/oyag146)

		Number of Patients		Median rwPFS (months)		Ribociclib vs Abemaciclib		
		Ribociclib	Abemaciclib	Ribociclib	Abemaciclib	HR	(95% CI)	
**Premenopausal State**	Yes	116	46	35	40	1.45	(0.83-2.52)	p = 0.191
	No	278	173	35	29	0.87	(0.66-1.15)	p = 0.324
**Age**	< 65	267	125	35	33	1.01	(0.74-1.37)	p = 0.970
	>/=65	127	94	36	29	0.88	(0.59-1.32)	p = 0.538
**PS**	ECOG 0	360	185	36	35	0.97	(0.74-1.26)	p = 0.813
	ECOG 1	34	34	19	26	1.35	(0.73-2.50)	p = 0.339
**Histology**	Ductal	294	160	35	35	1.13	(0.84-1.52)	p = 0.419
	Lobular	82	47	35	26	0.73	(0.45-1.17)	p = 0.186
**Ki67**	Low	190	114	36	30	0.99	(0.70-1.40)	p = 0.964
	High	204	105	35	36	0.95	(0.67-1.34)	p = 0.771
**Grading**	G1-G2	268	150	36	30	0.98	(0.73-1.32)	p = 0.907
	G3	126	69	34	33	0.94	(0.62-1.42)	p = 0.763
**ER**	Low	252	120	36	28	0.79	(0.58-1.07)	p = 0.125
	High	142	99	35	NR	1.27	(0.85-1.90)	p = 0.239
**PR**	Low	191	118	31	29	1.02	(0.74-1.40)	p = 0.924
	High	203	101	37	35	0.95	(0.66-1.38)	p = 0.792
**HER2**	0	258	133	36	30	0.95	(0.70-1.29)	p = 0.739
	Low	136	86	33	35	1.01	(0.68-1.51)	p = 0.960
**Neo or Adjuvant Chemotherapy**	Yes	147	115	33	28	0.97	(0.69-1.36)	p = 0.869
	No	247	104	36	36	1.03	(0.72-1.48)	p = 0.860
**Adjuvant Endocrine Therapy**	Yes	203	166	33	28	0.93	(0.70-1.23)	p = 0.600
	No	191	53	36	NR	1.58	(0.90-2.77)	p = 0.115
**Bone-only disease**	Yes	208	118	40	35	0.87	(0.61-1.23)	p = 0.422
	No	186	101	27	28	1.05	(0.75-1.47)	p = 0.788
**Bone Metastasis Number**	Low	192	125	37	30	0.88	(0.63-1.23)	p = 0.456
	High	202	94	22	32	1.06	(0.74-1.51)	p = 0.762
**Visceral Metastasis**	Yes	161	97	30	28	1.05	(0.74-1.50)	p = 0.765
	No	233	122	37	35	0.91	(0.65-1.27)	p = 0.559
**Setting**	Endocrine Resistant	96	105	27	27	1.02	(0.70-1.48)	p = 0.923
	Endocrine Sensitive	298	114	36	36	1.09	(0.78-1.52)	p = 0.636
**Endocrine Therapy**	Aromatase Inhibitor	268	148	36	32	0.96	(0.71-1.29)	p = 0.761
	Fulvestrant	126	71	31	29	0.99	(0.66-1.50)	p = 0.971

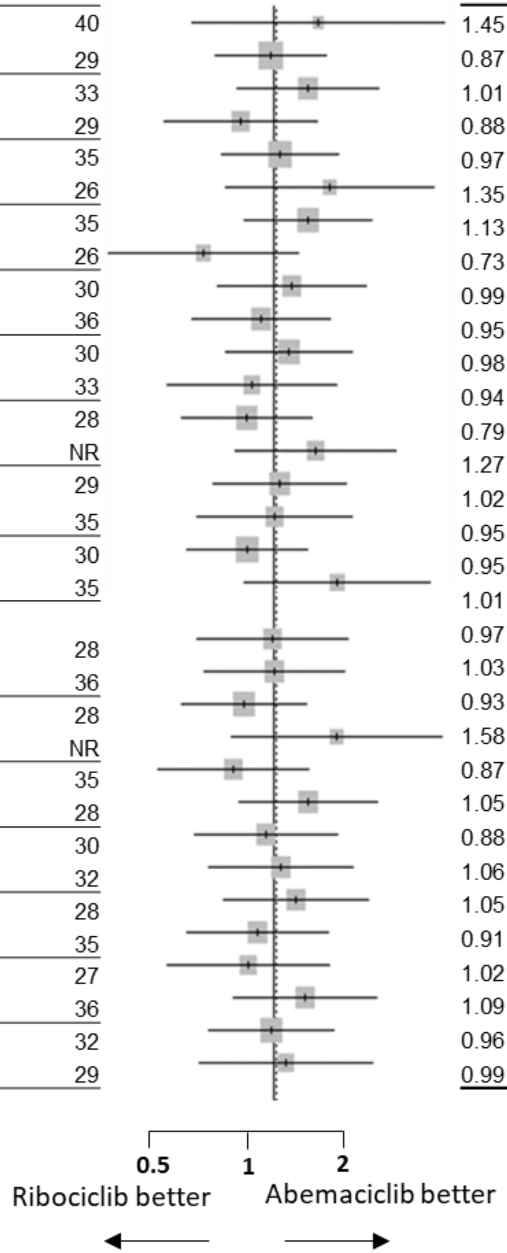

Supplement: oyag146_Supplementary_Data [file oyag146_supplementary_data.zip › Supplementary Figure 3.pdf]
