# Supplementary figures and images for: Comparative real-world progression free survival of CDK4/6 inhibitors in HR+/HER2− breast cancer patients with bone metastases
Source: Oncologist. 2026 Apr 16;31(5):oyag146. doi: 10.1093/oncolo/oyag146 (PMC13127761; doi:10.1093/oncolo/oyag146)

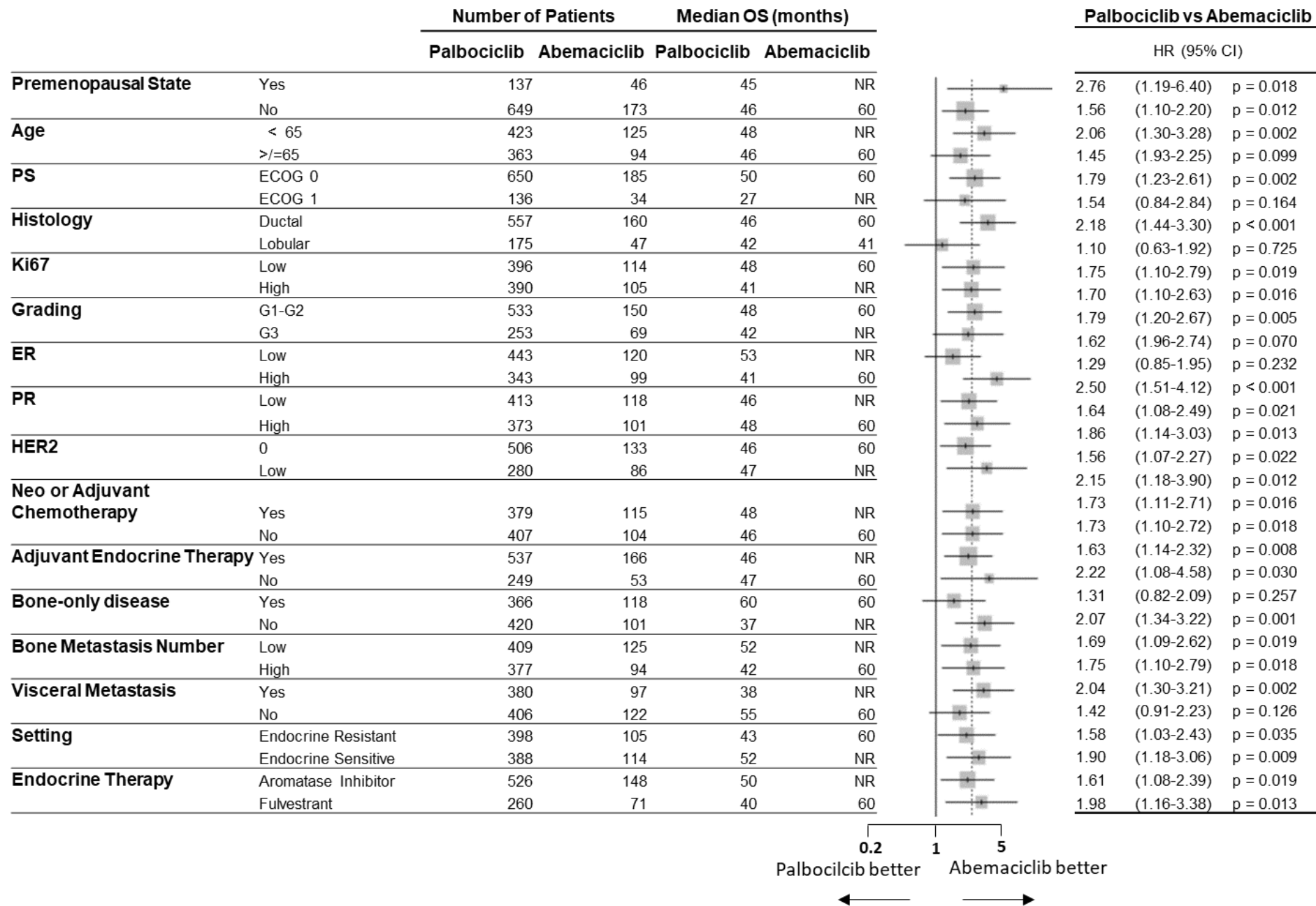

Supplement: oyag146_Supplementary_Data [file oyag146_supplementary_data.zip › Supplementary Figure 4.pdf]

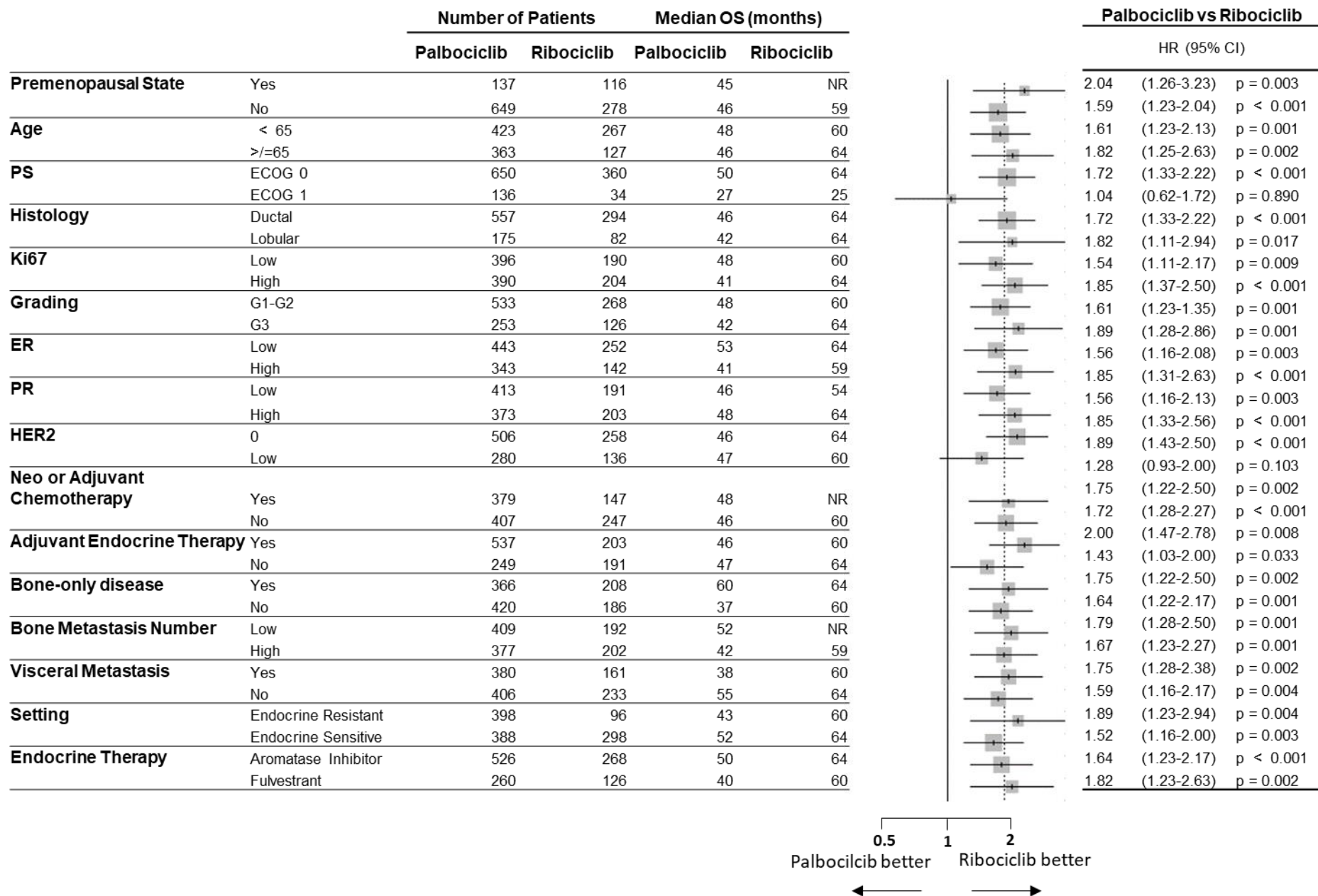

Supplement: oyag146_Supplementary_Data [file oyag146_supplementary_data.zip › Supplementary Figure 5.pdf]

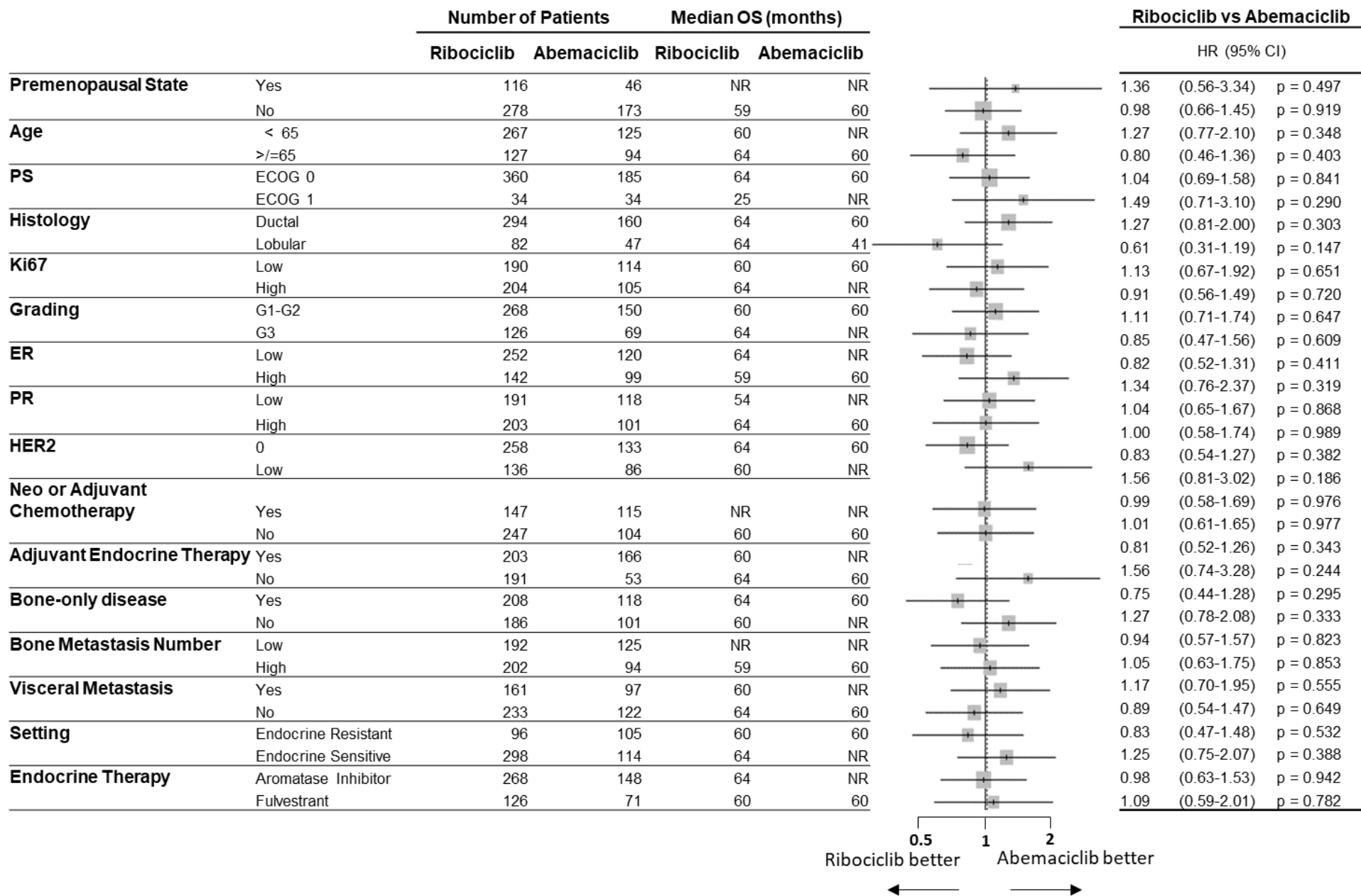

Supplement: oyag146_Supplementary_Data [file oyag146_supplementary_data.zip › Supplementary Figure 6.pdf]
